# Chromatography columns packed with thermoresponsive-cationic-polymer-modified beads for therapeutic drug monitoring

**DOI:** 10.1038/s41598-022-16928-3

**Published:** 2022-07-27

**Authors:** Kenichi Nagase, Hikaru Takagi, Hideo Nakada, Haruki Ishikawa, Yoshiko Nagata, Tohru Aomori, Hideko Kanazawa

**Affiliations:** 1 Faculty of Pharmacy, Keio University, 1-5-30 Shibakoen, Minato, Tokyo, 105-8512 Japan; 2 Department of Pharmacy, Keio University Hospital, 35 Shinanomachi, Shinjuku, Tokyo, 160-8582 Japan

**Keywords:** Analytical chemistry, Green chemistry

## Abstract

Therapeutic drug monitoring, which is used to determine appropriate drug doses, is critical in pharmacological therapy. In this study, we developed thermoresponsive chromatography columns with various cationic properties for effective therapeutic drug monitoring. Thermoresponsive cationic copolymer poly(*N*-isopropylacrylamide-*co*–*n*-butyl methacrylate-*co*–*N,N*-dimethylaminopropyl acrylamide) (P(NIPAAm-*co*-BMA-*co*-DMAPAAm))-modified silica beads, which were used as the chromatographic stationary phase, were prepared by modifying the radical initiator of the silica beads, followed by radical polymerization. Characterization of the prepared silica beads demonstrated that thermoresponsive polymers with various cationic properties successfully modified the beads. The elution behavior of several steroids in the prepared bead-packed columns at various temperatures indicated that the optimal column operating temperature was 30 °C. Appropriate measurement conditions for 13 drugs were investigated by varying the cationic properties of the columns and the pH of the mobile phase. Drug concentrations in serum samples were determined using the developed columns and mobile phases with a suitable pH. Voriconazole concentrations in human serum samples were determined using the developed columns with all-aqueous mobile phases. We anticipate that the developed chromatography columns can be used for therapeutic drug monitoring because drug concentrations can be measured using all-aqueous mobile phases that are suitable in clinical settings.

## Introduction

Therapeutic drug monitoring (TDM) for evaluating drug concentrations in patient serum samples is an effective method in pharmacological therapy^1^. Drug administration rate is not directly reflected by drug concentration in human serum samples owing to individual variations^1^. Therefore, maintaining a specific drug concentration in serum is critical, because low drug concentrations are ineffective, whereas excessively high drug concentrations can be toxic. Various types of TDM methods have been investigated, such as immunological methods based on enzyme-linked immunosorbent assay and chromatographic methods^2–4^. Liquid chromatography is a versatile method for evaluating drug concentration in serum samples because it does not require specific ligands for analytes^5^. Reversed-phase chromatography, which is a powerful method for determining drug concentrations in serum samples, requires an organic solvent in the mobile phase to modulate analyte retention on columns. However, several organic solvents used in the chromatographic mobile phase are toxic. Therefore, the use of organic solvents in hospitals should be avoided to prevent exposure.

Temperature-responsive chromatography using thermoresponsive polymers, such as poly(*N*-isopropylacrylamide) (PNIPAAm), can overcome this shortcoming because the chromatography system does not use organic solvents in the mobile phase^6–8^.

PNIPAAm, one of the most widely used thermoresponsive polymers, exhibits temperature-dependent hydrophobicity. This is attributed to the hydration and dehydration of PNIPAAm across its phase-transition temperature^9^. In addition, PNIPAAm extends and shrinks at low and high temperatures, respectively. Owing to its properties, PNIPAAm has been used for various biomedical applications, such as temperature-modulated drug delivery systems^10–15^, biosensors and bioimaging systems^16–21^, bioseparation systems^22–24^, nano-actuators^25–28^, temperature-modulated cell separation systems^29–35^, and cell culture substrates for regenerative medicine^36–40^.

Moreover, PNIPAAm-modified stationary phases have been used in chromatographic systems. The hydrophobicity of PNIPAAm in the stationary phase changes with column temperature, leading to modulation of the hydrophobic interactions between the stationary phase and analytes. Therefore, thermoresponsive chromatography does not require the use of organic solvents to modulate analyte retention.

In a previous study, a temperature-responsive chromatography column was investigated for applicability in TDM^41,42^. Drug concentrations in a sample can be analyzed using temperature-responsive chromatography with all-aqueous mobile phases^41,42^. However, several analytes have ionic properties and are not effectively retained on the column through only hydrophobic interactions.

In contrast, temperature-responsive ion-exchange chromatography has been used to separate ionic compounds through electrostatic and hydrophobic interactions^43–47^. Thermoresponsive ionic copolymers used for temperature-responsive ion-exchange chromatography are used to modify the stationary phase. In addition to hydrophobic interactions, electrostatic interactions between the ionic copolymers in the stationary phase and analytes can promote analyte separation. Therefore, temperature-responsive ion-exchange chromatography can be used to separate ionic analytes from all-aqueous mobile phases. Most drugs that require therapeutic monitoring exhibit both hydrophobic and ionic properties; thus, temperature-responsive ion-exchange chromatography is an effective TDM method.

**Figure 1:**
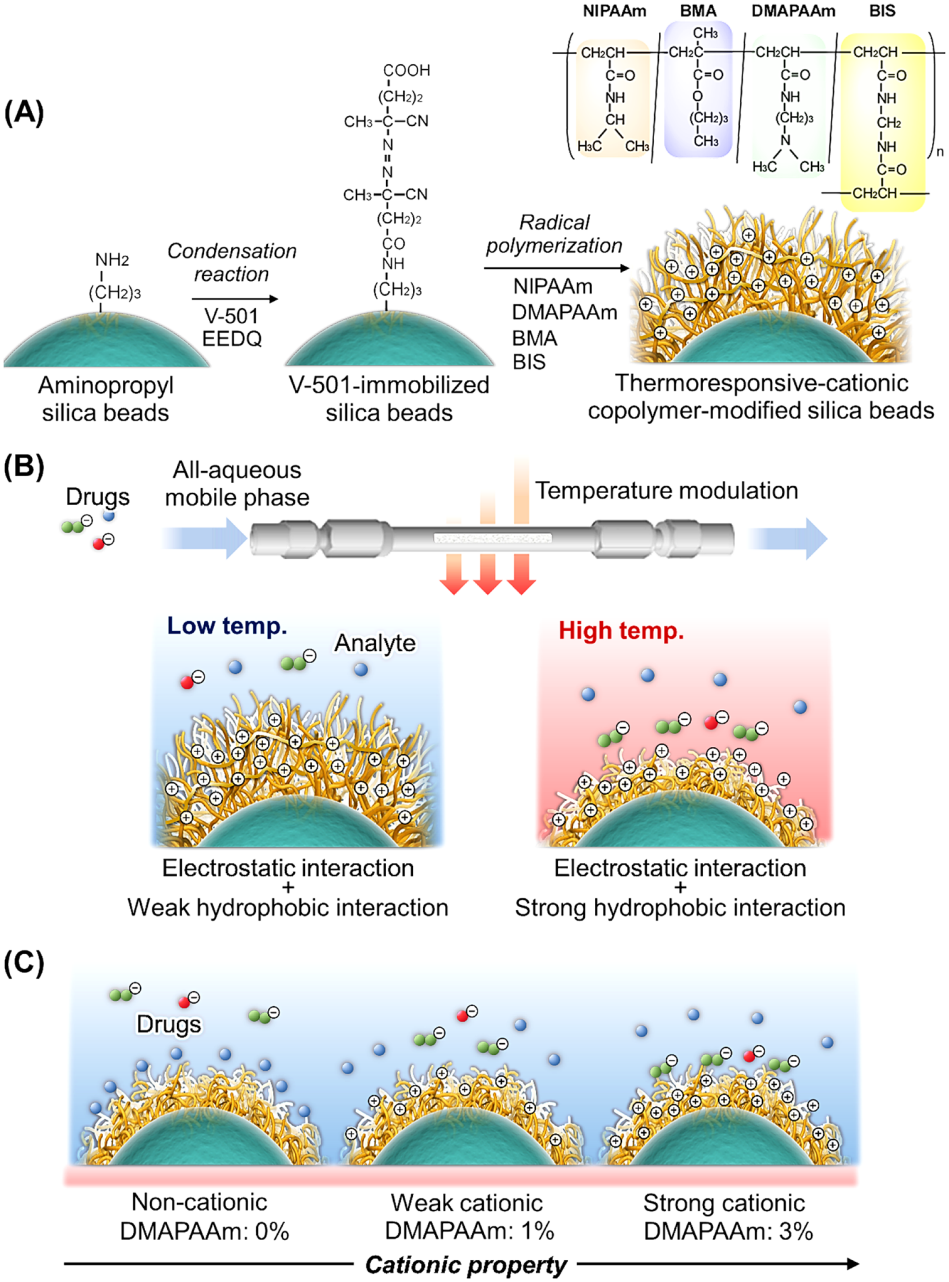
Thermoresponsive anion-exchange chromatography for therapeutic drug monitoring. (**A**) Schematic diagram of the preparation of a thermoresponsive cationic hydrogel. (**B**) Temperature-responsive property change of the modified copolymer on silica beads. (**C**) Electrostatic and hydrophobic interactions between the thermoresponsive cationic hydrogel and drugs in a chromatography column.

In this study, we developed a temperature-responsive ion-exchange chromatography method for TDM (Fig. 1). Silica beads modified with a thermoresponsive cationic copolymer were used as the stationary phase. *N,N*-dimethylaminopropyl acrylamide (DMAPAAm) was used as a cationic comonomer because it has a basic dimethylamino group with an acrylamide monomer. Previous reports have indicated that the PNIPAAm copolymer with cationic DMAPAAm exhibited electrostatic interactions with acidic biomolecules^44,48^. *n*-Butyl methacrylate (BMA) was used as a hydrophobic monomer because the incorporation of DMAPAAm into the PNIPAAm copolymer increased the phase transition temperature of the copolymer; this temperature should be modulated by incorporating hydrophobic monomers such as BMA^44^.

Using the prepared columns, the elution behavior of 13 drugs that require TDM (voriconazole, disopyramide, zonisamide, lidocaine, mexiletine, quinidine, carbamazepine, phenytoin, diazepam, lamotrigine, phenobarbital, mycophenolic acid, and sotalol) was observed to determine the optimal measurement conditions. The drug concentrations in serum samples were determined using the optimized measurement conditions. In addition, drug concentrations in clinical serum samples were determined using the temperature-responsive chromatography columns developed in this study.

## Results and discussion

### Characterization of the prepared copolymer-modified silica beads

Polymer-modified silica beads were prepared via a condensation reaction using 4,4ʹ-azobis(4-cyanovaleric acid) (V-501) as the initiator, followed by radical polymerization (Fig. 1A). The beads were prepared using P(NIPAAm-*co*-BMA-*co*-DMAPAAm) copolymers and are denoted as PN-0, PNBD-1, and PNBD-3 according to their initial monomers (*N*-isopropylacrylamide [NIPAAm], *n*-butyl methacrylate [BMA], and *N,N*-dimethylaminopropyl acrylamide [DMAPAAm], respectively) and feed ratios of the cationic monomer DMAPAAm. The feed ratios of the monomers are listed in Table 1, and the details of the fabrication procedure are included in the Methods section. The prepared copolymer-modified silica beads were characterized using CHN elemental analysis, Fourier-transform infrared (FT-IR) spectroscopy, zeta potential measurements, and scanning electron microscopy (SEM).

**Table 1:** Properties of the initiator- and polymer-modified beads. The samples are abbreviated using the initials of the monomers used to fabricate them (i.e., NIPAAm, BMA, and DMAPAAm) and their DMAPAAm content. The carbon content was estimated using elemental analysis, and the values are expressed as means ± standard deviations (n = 3). The %C_(calcd)_ values were calculated as the theoretical carbon contents of the initiator and polymers of the molecules. The amounts of initiator and polymer grafted onto the silica beads were calculated using their respective carbon content. The zeta potentials were measured using a zeta potential analyzer, and the values are expressed as means ± standard deviations (n = 3).

Sample	Monomer feed ratio (%)			Elemental composition (%)		%C_(calcd)_	Modified initiator (μmol/m^2^)	Grafted polymer (mg/m^2^)	Zeta potential (mV)	
	NIPAAm	BMA	DMAPAAm	C	N				10 °C	30 °C
Aminopropyl silica beads				3.21 ± 0.02	1.32 ± 0.05				14.30 ± 1.04	13.63 ± 0.70
Initiator V-501-modified beads				9.32 ± 0.03	3.12 ± 0.01	51.37	1.55		− 18.93 ± 0.31	− 10.43 ± 0.40
PN-0	100	0	0	12.94 ± 0.07	3.19 ± 0.05	63.63		0.223	− 8.58 ± 0.24	− 4.46 ± 0.54
PNBD-1	94	5	1	13.84 ± 0.08	3.40 ± 0.13	63.80		0.282	− 6.16 ± 0.58	− 3.58 ± 0.40
PNBD-3	92	5	3	14.95 ± 0.03	3.68 ± 0.03	63.75		0.359	0.97 ± 0.65	4.47 ± 0.30

The carbon and nitrogen contents of the beads obtained under different reaction conditions were measured using elemental analysis (Table 1). The carbon and nitrogen contents of the V-501-modified beads were higher than those of the aminopropyl silica-modified beads. This indicates that V-501 successfully modified the beads through a condensation reaction between V-501 and the aminopropyl groups of the silica beads, which led to an increase in the carbon and nitrogen contents of the beads. The amount of V-501 on the surface of the silica beads was 1.55 μmol/m^2^, which was comparable to that of the previously reported V-501-modified silica beads obtained under similar reaction conditions^49,50^. Moreover, the carbon and nitrogen contents of the PN-0, PNBD-1, and PNBD-3 beads in this study were higher than those of the V-501-modified beads. These results indicate that the copolymers successfully modified the silica beads via radical polymerization. The amount of modified copolymer increased as follows: PN-0 < PNBD-1 < PNBD-3. This may have been due to the molecular weights of BMA and DMAPAAm being larger than those of NIPAAm, or the reactivities of BMA and DMAPAAm being higher than that of NIPAAm.

**Figure 2:**
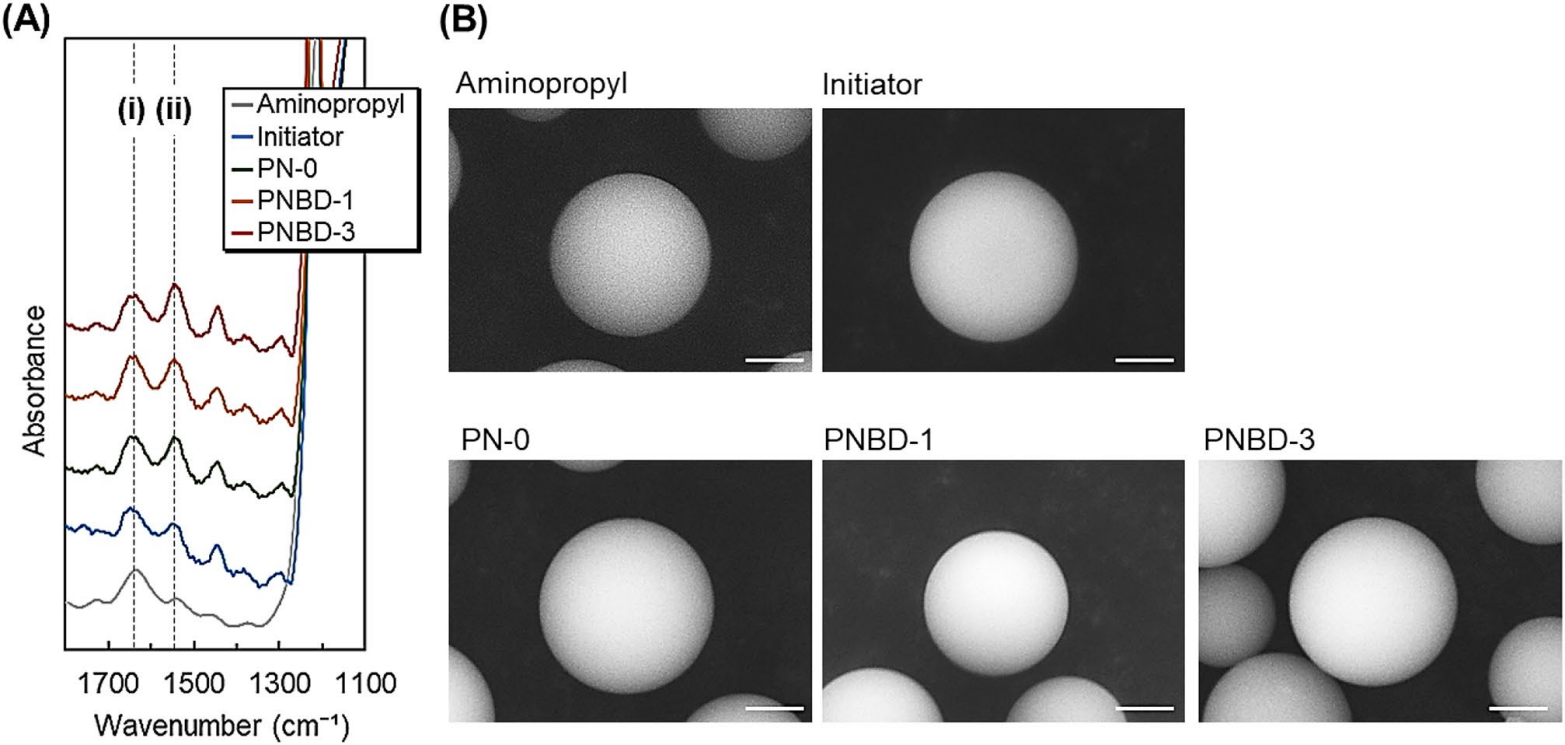
(**A**) FT-IR spectra and (**B**) SEM images of the prepared beads. Scale bar: 2 μm.

FT-IR spectra of the prepared beads were also obtained to confirm that the copolymers modified the silica beads (Fig. 2A). Peaks at approximately 1550 and 1645 cm^-1^ in the FT-IR spectra of the copolymer-modified beads (PN-0, PNBD-1, and PNBD-3) were observed, which were attributed to the amide bonds of the polymers. In contrast, the peaks of the aminopropyl silica beads were relatively low compared with those of the copolymer-modified silica beads. These results indicate that the three types of copolymers were successfully modified on silica beads through radical polymerization. The absorbance of aminopropyl silica beads increased with increasing wavenumber, which led to a small difference in the peak height between the copolymer-modified silica beads and aminopropyl silica beads compared with that of the previously reported FT-IR analysis of the polymer-modified beads^41^.

In this study, the actual composition of the monomer in the copolymer could not be directly measured by nuclear magnetic resonance because the copolymer was modified on silica beads by surface-initiated radical polymerization. A previous report indicated that the monomer composition of the modified P(NIPAAm-*co*-BMA-*co*-DMAPAAm) on silica beads is almost the same as the monomer feed composition in radical polymerization^31^. Thus, in the present study, the actual monomer composition of the modified copolymer would be approximately the same as the monomer feed composition.

Phase transition temperatures of copolymers of similar composition were measured in previous studies^31^. The phase transition temperature of PNIPAAm (the same composition of PN-0) is 32.7 °C^51^. The phase transition temperature of P(NIPAAm-*co*-BMA-*co*-DMAPAAm) with the composition of 5 mol% BMA and 1 mol% DMAPAAm (the same composition of PNBD-1) is 22.5 °C^31^. The phase transition temperature of P(NIPAAm-*co*-BMA-*co*-DMAPAAm) with the composition of 5 mol% BMA and 5 mol% DMAPAAm (similar composition to PNBD-3, with slightly more DMAPAAm) is 27.5 °C^31^. Thus, the phase transition temperature of the modified copolymer on silica beads in this study would be similar to these reported temperatures.

The zeta potentials of the prepared beads were determined to investigate their electrostatic properties (Table 1). The cationic properties of the aminopropyl silica-modified beads were attributed to their amino groups. Moreover, the anionic properties of the V-501-modified silica beads were attributed to the carboxyl groups of the V-501 molecules present on the surface of the beads. The zeta potentials of the PN-0, PNBD-1, and PNBD-3 beads were higher than those of the V-501-modified silica beads because of the modification of the beads through the reaction of V-501 to initiate polymerization. Moreover, the zeta potentials of the PN-0, PNBD-1, and PNBD-3 beads increased in the following order: PN-0 < PNBD-1 < PNBD-3; this was because the amount of cationic monomer DMAPAAm incorporated into the copolymers increased in the same order. A slightly higher zeta potential of cationic copolymer-modified silica beads was observed at 30 °C than at 10 °C. This was likely because the dehydration of the modified polymer on the silica beads tended to aggregate, and the electrophoretic velocity of the polymer-modified beads was reduced.

The bead morphology after each reaction was observed using SEM (Fig. 2B). The beads retained their spherical shape, regardless of the reaction conditions. These results indicate that the V-501 immobilization and polymer modification reactions did not deform the silica beads.

### Elution behavior of steroids in the bead-packed columns

The prepared polymer-modified beads were packed into stainless-steel columns (column diameter, 4.6 mm; column length, 50 mm). The columns were connected to a high-performance liquid chromatography (HPLC) system, and the elution behavior of several analytes was observed to investigate the performance of the columns packed with the prepared beads for monitoring the drug concentrations in serum samples.

**Figure 3:**
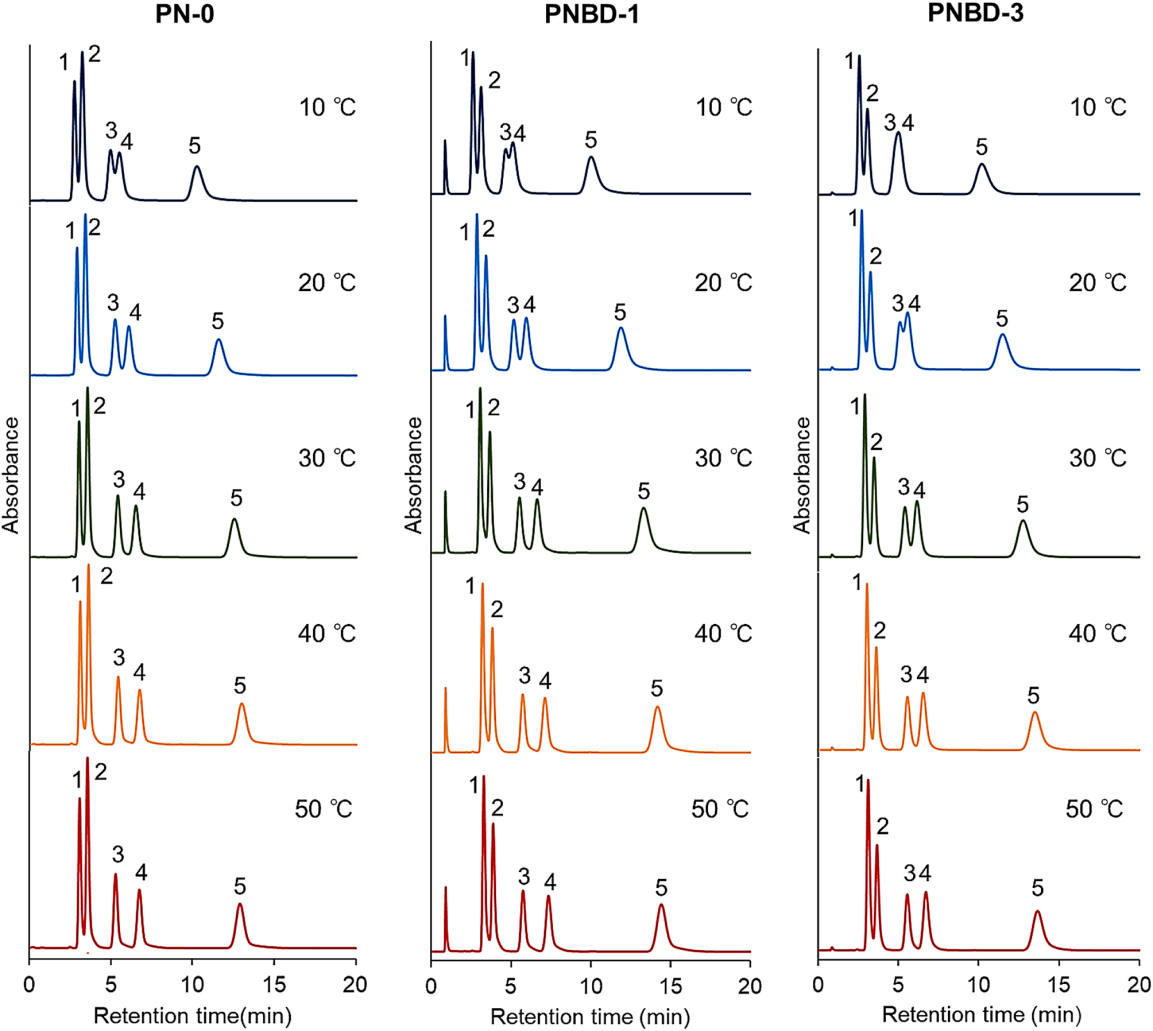
Chromatograms of steroids obtained using the prepared thermoresponsive-cationic-polymer-hydrogel-modified silica beads. Mobile phase: pure water; mobile phase flow rate: 1.0 mL/min; detection wavelength: 254 nm. Peaks 1: hydrocortisone, 2: prednisolone, 3: dexamethasone, 4: hydrocortisone acetate, and 5: testosterone.

First, the elution behavior of several hydrophobic steroids was observed at various column temperatures (Fig. 3) to investigate the hydrophobic properties of the prepared columns and thermoresponsive properties of the stationary phases. The properties of the hydrophobic steroids are presented in Table S1. The changes in the retention times of the hydrophobic steroids with column temperature are illustrated in Fig. S1. For all columns, the retention times of the hydrophobic steroids increased with increasing column temperature. This was ascribed to the copolymers used to modify the silica beads, which dehydrated and became hydrophobic with increasing temperature, leading to an enhancement in the hydrophobic interactions between the polymers and steroids. The retention times of the hydrophobic steroids in the PNBD-1 column were longer than those in the PN-0 and PNBD-3 columns. This was attributed to the stronger hydrophobicity of the copolymer in the PNBD-1 column. Previous reports have indicated that the hydrophobicity of PNIPAAm copolymers depends on the amount of incorporated monomers^7,52–54^, and increases with increasing BMA content^55,56^. Conversely, the hydrophobicity of the PNIPAAm copolymers decreases with increasing ionic monomer content^45,57–59^. The PNBD-1 and PNBD-3 copolymers contained hydrophobic BMA, which imparted hydrophobicity; however, the copolymers also contained cationic DMAPAAm, conferring hydrophilicity to the copolymers. Therefore, the hydrophobicities of the PNBD-1 and PNBD-3 copolymers were determined by balancing these factors. The DMAPAAm content of PNBD-3 was higher than that of PNBD-1; therefore, PNBD-3 was less hydrophobic than PNBD-1. Moreover, PN-0 comprised NIPAAm only, whereas PNBD-1 contained both hydrophobic BMA and hydrophilic cationic DMAPAAm. The BMA and DMAPAAm contents of the feed used to fabricate PNBD-1 were 5% and 1%, respectively. Therefore, owing to its relatively high content of BMA, PNBD-1 was hydrophobic. These factors caused the retention times of steroids on the PNBD-1 column to be longer than those on the PN-0 and PNBD-3 columns. The peaks of dexamethasone and hydrocortisone acetate became separated with increasing column temperature on all columns, and these steroids were almost completely separated at 30 °C (Fig. 3). In contrast, an excessively high column temperature would promote the hydrolysis of silica beads, causing their stability to decrease^60^. Therefore, 30 °C was the optimal temperature for these columns.

### Elution behavior of drugs under various experimental conditions

The elution behavior of 13 drugs that require TDM was observed to investigate the applicability of the developed columns for TDM. The properties of these drugs are summarized in Tables S2-1 and S2-2. The elution behavior of each drug was observed on the PN-0, PNBD-1, and PNBD-3 columns using mobile phases with pH 4.7 and 6.8. The elution behavior of the drugs and their retention times and peak areas are presented in Figs. S2–S14, and the experimental data are summarized in Tables S3–S15.

In TDM, a long retention time is not desirable, because the drug concentration in the blood should be promptly determined for optimized drug administration. The contaminant peak from the serum was observed at a retention time of approximately 1 min because the serum was not retained in the PNIPAAm copolymer^41^. The drug peak should be separated from the contaminant peak; thus, a drug peak separated from the contaminant peak and at a short retention time is desirable. In addition, the small peak area owing to adsorption onto the column was not desirable for measuring the drug concentration. Thus, optimal measurement conditions were determined based on the proper retention time and peak area.

The elution behaviors of voriconazole and zonisamide are presented in Figs. S2 and S3, respectively, and their corresponding retention times and peak areas are summarized in Tables S3 and S4, respectively. The elution behaviors of voriconazole and zonisamide using mobile phases with pH 4.7 and 6.8 were comparable, indicating that the retention of voriconazole and zonisamide was not significantly affected by the pH of the mobile phase. Similar elution behaviors were observed for all three types of columns, indicating that the hydrophobicity and cationic properties of the copolymers that modified the packed beads did not significantly affect the retention of voriconazole and zonisamide. Accordingly, the PN-0 column using the mobile phase with a pH of 4.7 was suitable for monitoring voriconazole and zonisamide.

The elution behavior of diazepam is shown in Fig. S4, and its retention times and peak areas are summarized in Table S5. The elution behavior of diazepam did not change with the pH of the mobile phase (4.7 or 6.8), indicating that the pH of the mobile phase did not affect diazepam retention. In addition, the elution behavior of diazepam on the three types of columns was similar, indicating that slight changes in the hydrophobic and cationic properties of the copolymers used to modify the silica beads did not affect diazepam retention. Moreover, the retention time of diazepam was longer than that of the other drugs. Excessively long retention times are not suitable in TDM because they lead to long measurement times, therefore preventing prompt determination of drug levels. Accordingly, the PN-0 column using the mobile phase with a pH of 6.8 was the most suitable for monitoring diazepam because the diazepam retention time on this column was the shortest.

The elution behavior of lamotrigine is shown in Fig. S5, and its retention times and peak areas are summarized in Table S6. Lamotrigine elution was ineffective in the columns using the mobile phase with a pH of 4.7. However, a sharp peak with an adequate retention time was observed in the columns using the mobile phase with a pH of 6.8. This was ascribed to the p*K*_a_ of lamotrigine (5.70). Therefore, lamotrigine adopted molecular and ionic forms in the columns using mobile phases with pH 4.7 and 6.8, respectively. This contributed to the difference in the elution behavior of lamotrigine in the columns using mobile phases with different pH values. In summary, the PNBD-3 column using the mobile phase with a pH of 6.8 was optimal for monitoring lamotrigine owing to the short retention time and large peak area of lamotrigine on this column.

The elution behavior of phenytoin is illustrated in Fig. S6, and its retention times and peak areas are summarized in Table S7. The retention times of phenytoin in the cationic columns, PNBD-1 and PNBD-3, were longer than that in the non-cationic column, PN-0. This was ascribed to the interaction of acidic phenytoin with the cationic copolymers, PNBD-1 and PNBD-3. Similar peak areas were obtained, regardless of the experimental conditions. The PN-0 column using the mobile phase with a pH of 6.8 was optimal for phenytoin monitoring because the retention time of phenytoin on this column was the shortest.

The elution behavior of phenobarbital is summarized in Fig. S7, and its retention times and peak areas are summarized in Table S8. The peak areas of phenobarbital at pH 4.7 were smaller than at pH 6.8, because phenobarbital adopted a molecular form with decreasing mobile phase pH, causing its adsorption onto the stationary phase. Our experimental results indicated that the PNBD-3 column using the mobile phase with a pH of 6.8 was optimal for phenobarbital monitoring.

The elution behavior of carbamazepine is summarized in Fig. S8, and its retention times and peak areas are summarized in Table S9. The peak area of carbamazepine in the PN-0 column using the mobile phase with a pH of 4.7 was the smallest. This was ascribed to the stronger adsorption of carbamazepine on the PN-0 polymer at pH 4.7 than at pH 6.8. Therefore, the PN-0 column using the mobile phase with a pH of 6.8 was optimal for carbamazepine monitoring because the retention time and peak area of carbamazepine on this column were short and large, respectively.

The elution behavior of sotalol is shown in Fig. S9, and its retention times and peak areas are summarized in Table S10. Owing to its basic properties, sotalol was retained only on the PN-0 and PNBD-1 columns using the mobile phase with a pH of 6.8. The retention times and peak areas of sotalol indicated that the PNBD-1 column using the mobile phase with a pH of 6.8 was more suitable for monitoring sotalol than the PN-0 column with the same conditions.

The elution behaviors of mexiletine, lidocaine, disopyramide, and quinidine are shown in Figs. S10, S11, S12, and S13, respectively, and their corresponding retention times and peak areas are summarized in TablesS11, S12, S13, and S14, respectively. The retention times of mexiletine, lidocaine, disopyramide, and quinidine increased in the following order: PNBD-3 < PNBD-1 < PN-0. Owing to their high p*K*_a_ values, these drugs adopted ionic forms in the presence of mobile phases with pH 4.7 and 6.8. Accordingly, owing to their basic properties, these drugs repelled the cationic groups of the PNBD-1 and PNBD-3 copolymers, decreasing their retention times on the cationic PNBD-1 and PNBD-3 columns. The retention times of these drugs in the columns using the mobile phase with a pH of 6.8 were longer than those in the columns using the mobile phase with a pH of 4.7. This was ascribed to the drugs adopting predominantly molecular forms with increasing mobile phase pH, leading to stronger hydrophobic interactions between drugs and copolymers. Considering the retention times and peak areas, the PN-0 column using the mobile phase with a pH of 4.7 was considered suitable for mexiletine, lidocaine, and disopyramide monitoring, whereas the PNBD-1 column using the mobile phase with a pH of 4.7 was suitable for quinidine monitoring.

The elution behavior of mycophenolic acid is illustrated in Fig. S14, and its retention times and peak areas are summarized in Table S15. The retention times of mycophenolic acid in the columns using the mobile phase with a pH of 6.8 were shorter than those in the columns using the mobile phase with a pH of 4.7. This was attributed to mycophenolic acid adopting a predominantly molecular form with decreasing mobile phase pH, leading to weaker hydrophobic interactions between mycophenolic acid and the polymers. The retention times of mycophenolic acid on the cationic columns, PNBD-1 and PNBD-3, using the mobile phase with a pH of 6.8 were longer than those on the PN-0 column. This was attributed to the electrostatic interactions between mycophenolic acid and the cationic groups of the PNBD-1 and PNBD-3 copolymers.

**Figure 4:**
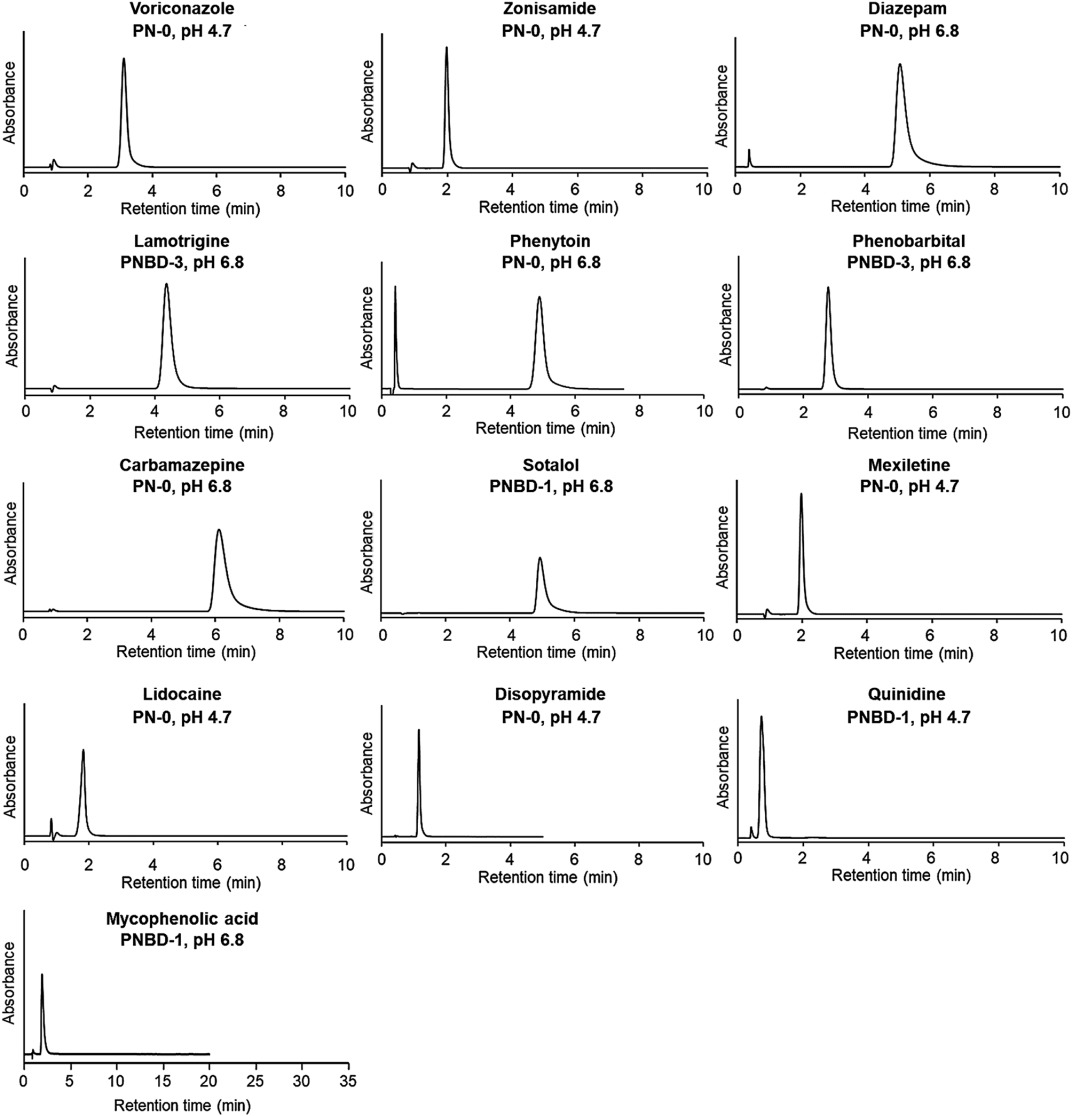
Chromatograms of drugs obtained using the suitable columns packed with thermoresponsive cationic-copolymer-modified silica beads. Mobile phase: 10 mmol/L CH_3_COONH_4_ (pH 4.7) or 10 mmol/L CH_3_COONH_4_ (pH 6.8); mobile phase flow rate: 1.0 mL/min. Detection wavelengths for each drug are summarized in Table S2.

The optimal measurement conditions for the 13 drugs used in this study are summarized in Table S16. The elution behaviors of the drugs under the optimal measurement conditions are presented in Fig. 4. Sharp peaks emerged in the chromatograms of all the drugs under the optimized measurement conditions.

Eight drugs (voriconazole, zonisamide, diazepam, phenytoin, carbamazepine, mexiletine, lidocaine, and disopyramide) were effectively retained on the PN-0 column owing to hydrophobic interactions between PNIPAAm and these drugs. Among them, voriconazole, zonisamide, mexiletine, lidocaine, and disopyramide were effectively retained on the column using the mobile phase with a pH of 4.7, likely because the ionization of the drugs was suppressed, leading to strong hydrophobic interactions between the drugs and PNIPAAm. Sotalol, quinidine, and mycophenolic acid were effectively retained on the relatively weak cationic PNBD-1 column. Lamotrigine and phenobarbital were retained on the relatively strong cationic PNBD-3 column. The cationic properties of the PNBD-1 and PNBD-3 columns led to suitable retention times and large peak areas of these drugs.

### Determination of drug concentration in serum samples

**Figure 5:**
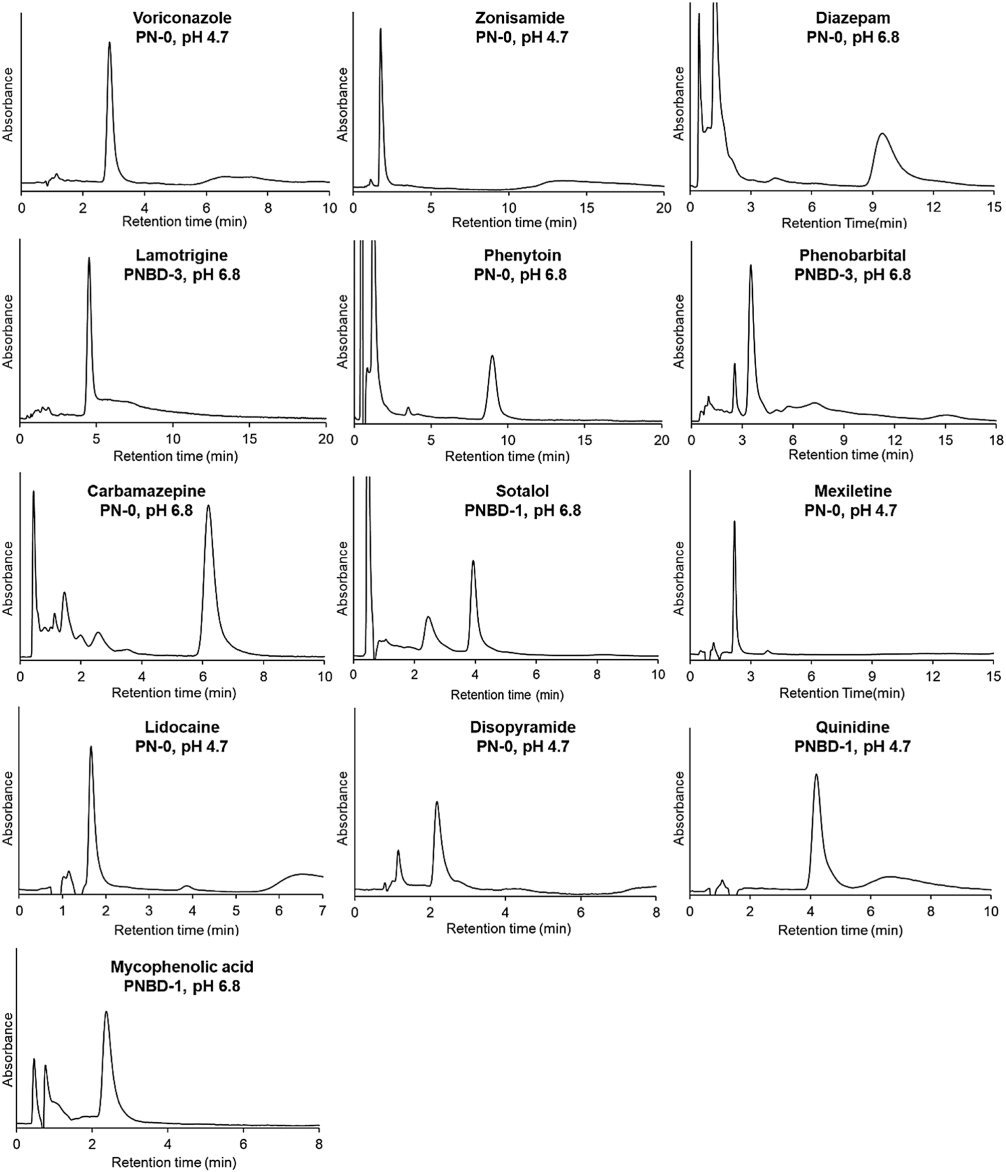
Chromatograms of the drugs with serum proteins obtained using the suitable columns packed with thermoresponsive cationic-copolymer-modified silica beads. Mobile phase: 10 mmol/L CH_3_COONH_4_ (pH 4.7) or 10 mmol/L CH_3_COONH_4_ (pH 6.8); flow rate: 1.0 mL/min. Detection wavelengths for each drug are summarized in Table S2.

**Figure 6:**
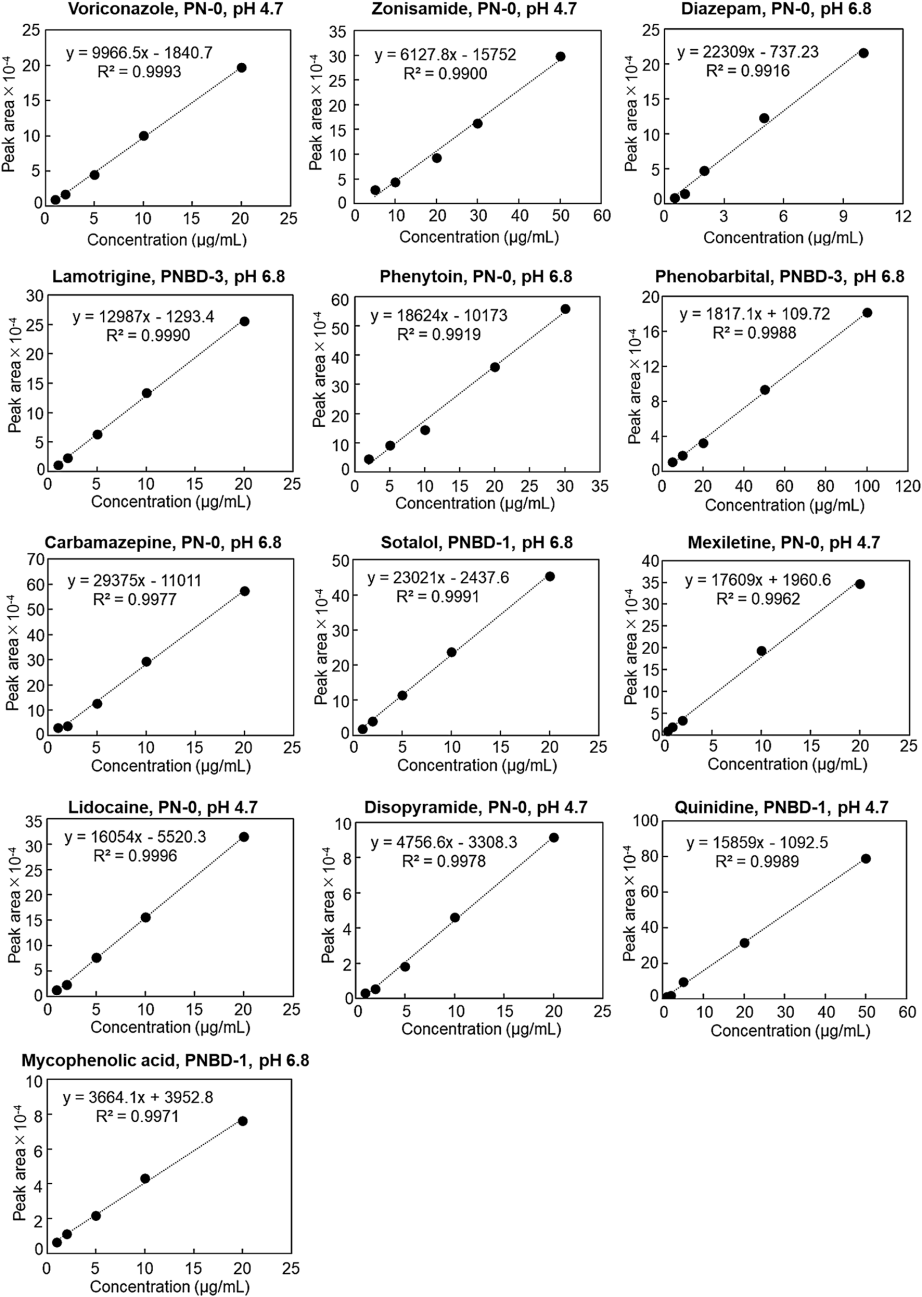
Calibration curves of the drugs with serum proteins (*n* = 5).

Drug concentrations in serum samples should be determined during TDM. Accordingly, the 13 drugs were dissolved in serum, and the prepared serum samples containing the drugs at various concentrations were used as model analytes for clinical samples. The prepared samples were pretreated before injection into the chromatography columns using a solid-phase extraction spin centrifuge column. The elution behavior of each serum drug sample was observed under optimized conditions (Fig. 5) and calibration curves were obtained at concentrations similar to the effective drug concentrations used for treatment (Fig. 6). Although a contaminant peak emerged in each chromatogram, which was ascribed to the serum components, the peaks used to determine the concentration of each drug were distinctly observed (Fig. 5). In addition, the calibration curves of the drugs were approximately linear near the effective drug concentration range (Fig. 6). The correlation coefficients of the calibration curves were high (> 0.99), indicating that the drug concentration in the serum samples could be determined using the developed columns with suitable mobile phases. The peak areas of the samples containing low concentrations of zonisamide (20 μg/mL) and phenytoin (10 μg/mL) were slightly different from those determined using linear approximations, leading to relatively low correlation coefficients (0.9900 and 0.9919 for zonisamide and phenytoin, respectively). The peak area of the sample with a diazepam concentration of 5 μg/mL was slightly different from that determined using linear approximation, leading to a relatively low correlation coefficient (0.9916). The calibration curves for the other 10 drugs showed high correlation coefficients (> 0.996).

**Figure 7:**
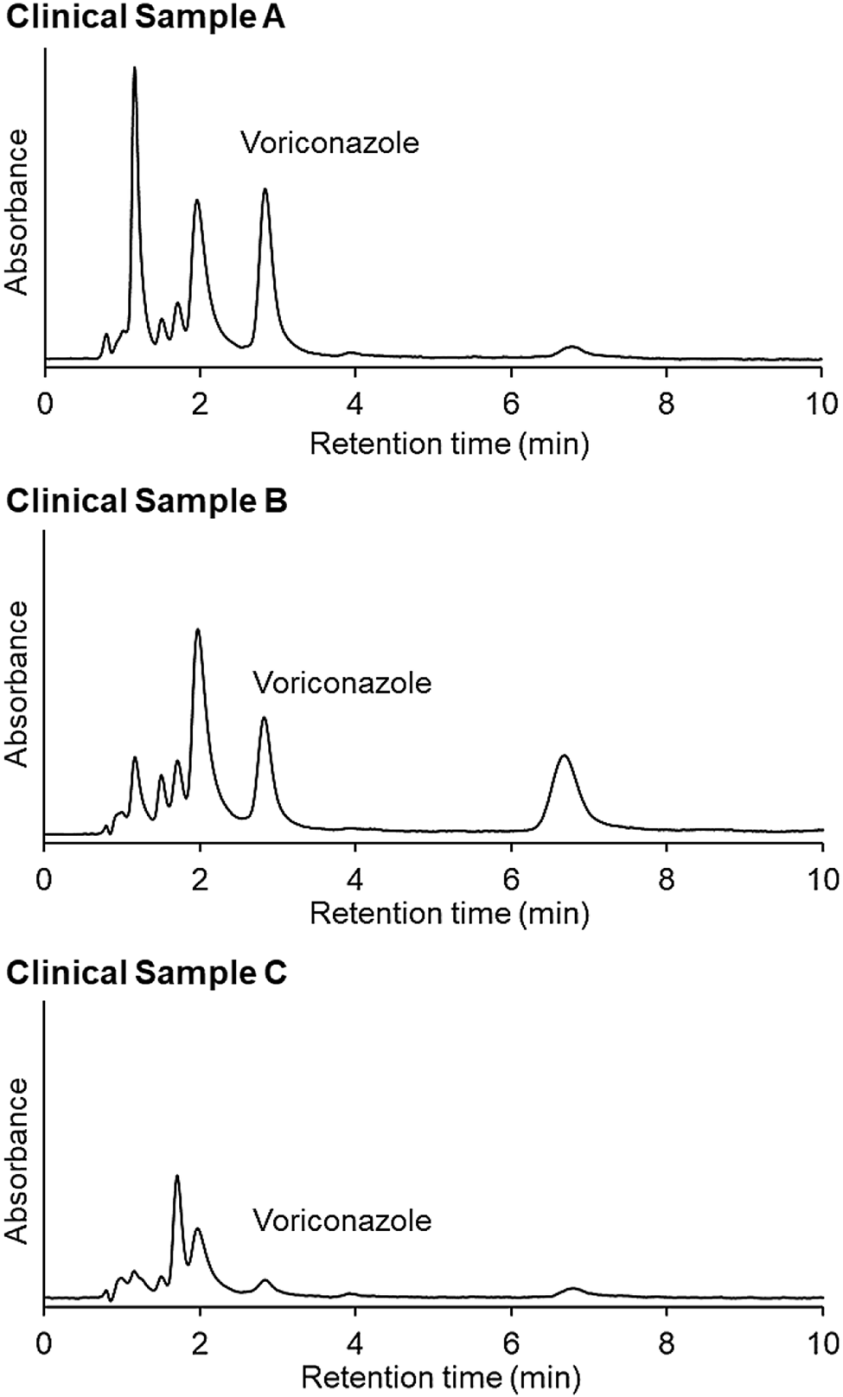
Chromatograms of clinical samples collected from patients who were administered voriconazole. Column: PN-0; mobile phase: 10 mmol/L CH_3_COONH_4_ (pH 4.7); mobile phase flow rate: 1.0 mL/min; detection wavelength: 250 nm.

Human serum samples collected from three patients who were administered voriconazole were analyzed using the PN-0 column with a mobile phase comprising 10 mmol/L CH_3_COONH_4_ (pH 4.7). The elution behavior of the serum samples is shown in Fig. 7. Voriconazole peaks were observed in the chromatograms of all the clinical samples. In addition, the relative standard deviations of the peak areas for the five repeated measurements were relatively low for samples A and B (Table 2), indicating that the developed methods presented good repeatability. The peak area of sample C was relatively low because the drug concentration in the serum sample was low, resulting in a relatively low standard deviation of the peak area. Additionally, the concentrations of voriconazole in the human serum samples were determined using the obtained calibration curve. The calculated voriconazole concentrations were comparable to those obtained using conventional reversed-phase chromatography (Table 2). These results demonstrate that the developed thermoresponsive chromatography columns have similar analytical efficiencies and can be used to determine drug concentrations in human serum samples.

**Table 2:** Retention times and peak areas of voriconazole in three clinical samples (*n* = 5).

Sample	PN-0 column					Reversed-phase column
	Retention time (min)	% RSD of the retention time	Peak area	% RSD of the peak area	Estimated concentration (µg/mL)	Estimated concentration (µg/mL)
Sample A	2.84	0.05	24,095	0.61	2.60	2.76
Sample B	2.82	0.06	15,838	0.93	1.77	1.87
Sample C	2.84	0.16	2251	3.01	0.41	0.32

The developed columns packed with thermoresponsive-cationic-polymer-modified beads can be used to monitor the concentrations of 13 drugs in serum samples using all-aqueous mobile phases. The stationary and mobile phases of the columns and the pH of the mobile phase should be carefully selected depending on the properties of each drug. Additionally, these columns can be used to determine the concentration of voriconazole in human serum samples. In summary, the proposed method can be used to determine drug concentrations in human serum samples using all-aqueous mobile phases, which is desirable for clinical practice. Therefore, the developed chromatography columns can be used for TDM.

## Conclusions

Thermoresponsive chromatography columns packed with thermoresponsive-cationic-polymer-modified beads were developed in this study for effective and useful TDM. Thermoresponsive cationic copolymers with various cationic properties were used to modify silica beads through radical polymerization. Elemental analysis and FT-IR spectroscopy confirmed that the copolymers successfully modified the silica beads. The zeta potentials of the prepared silica beads indicated that their cationic properties increased with increasing cationic monomer content. The temperature-dependent elution behavior of several steroids was attributed to copolymer dehydration. The elution behavior of several steroids indicated that 30 °C was a suitable column temperature. The elution behaviors of 13 drugs, which require TDM, were observed using three types of prepared columns and mobile phases with pH 4.7 and 6.8. Optimal measurement conditions for each drug were determined. Our results indicate that drug concentrations in serum samples can be determined using suitable columns and mobile phases. Moreover, drug concentrations in human serum samples were determined using the developed columns and all-aqueous mobile phases. The developed thermoresponsive chromatography columns with various cationic properties can be useful tools for TDM because drug concentrations can be monitored using all-aqueous mobile phases, which are suitable in medical settings.

## Methods

### Preparation of the thermoresponsive-cationic-polymer-modified beads

Polymer-modified silica beads, which were used as packing materials, were prepared by initiator modification via condensation, followed by radical polymerization of the functional monomers for polymer modification (Fig. 1A)^61–63^. First, *N*-ethoxycarbonyl-2-ethoxy-1,2-dihydroquinoline (6.18 g, 25.0 mmol) and V-501 (3.50 g, 12.5 mmol) were dissolved in 25 mL of *N,N*-dimethylformamide (DMF) in a 100 mL flask. Aminopropyl silica beads (5.0 g) were added to the solution and DMF was added to the suspension to a total suspension volume of 50 mL. The suspension was deoxygenated by bubbling with nitrogen gas for 30 min under sonication in an ultrasonic bath. Thereafter, the flask was sealed and the V-501 modification reaction was allowed to proceed at 25 °C for 6 h under continuous flask shaking using a shaker. Subsequently, the beads were filtered, rinsed with ethanol, and dried under vacuum.

Thermoresponsive cationic polymers with various compositions were used to modify silica beads via radical polymerization. To fabricate the PNBD-3 beads, NIPAAm (4.50 g, 39.8 mmol), BMA (0.307 g, 2.16 mmol), DMAPAAm (0.202 g, 1.29 mmol), and *N,N'*-methylene*bis*acrylamide were dissolved in 50 mL of ethanol in a 200 mL flask. Next, V-501–modified silica beads (2.00 g) were added to the mixture, and ethanol was added to a total volume of 100 mL. Thereafter, the suspension was deoxygenated by bubbling with nitrogen gas for 30 min under sonication. Subsequently, the flask was sealed, and polymerization was performed at 70 °C for 5 h under continuous shaking using a shaker. After polymerization, the beads were filtered, rinsed with methanol, and dried under vacuum.

### Characterization of the polymer-modified beads

The polymer-modified silica beads were characterized by elemental analysis, FT-IR spectroscopy, zeta potential measurements, and SEM. CHN elemental analysis of the silica beads was performed using an elemental analyzer (PE-2400; PerkinElmer, Waltham, MA, USA) to determine the amounts of the modifying initiator and polymer on the bead surfaces. The amount of modified V-501 on the silica beads was estimated using the carbon composition of the beads obtained through elemental analysis, as follows:

1 $$\frac{{\% C_{I} }}{{\% C_{I} \left( {calcd} \right) \times \left( {1 - \% C_{I} /\% C_{I} \left( {calcd} \right)} \right) \times S}}$$

where *%C*_*I*_ is the increase in the carbon content of the beads after V-501 modification, %*C*_*I*_ (*calcd*) is the calculated carbon content of the V-501 molecules, and *S* is the surface area of the aminopropyl silica beads (310 m^2^/g).

The amount of polymers that modified the silica beads was obtained as follows:

2 $$\frac{{\% C_{P} }}{{\% C_{P} \left( {calcd} \right) \times \left( {1 - \% C_{P} /\% C_{P} \left( {calcd} \right) - \% C_{I} /\% C_{I} \left( {calcd} \right)} \right) \times S}}$$

where *%C*_*P*_ is the increase in the carbon content of the beads after polymer modification and %*C*_*P*_ (*calcd*) is the calculated carbon content of each polymer.

Polymer modification of the beads was confirmed using their FT-IR spectra. The spectra of the beads were obtained using an attenuated total reflection–FT-IR spectrometer (FT/IR-4700; JASCO, Tokyo, Japan).

To investigate the cationic properties of the prepared beads, their zeta potentials were measured using a zeta potential analyzer (Zetasizer Nano-ZS; Malvern Panalytical, Malvern, UK). Copolymer-modified beads were suspended in a 5 mmol/L KCl solution at a bead concentration of 0.5 mg/mL. The prepared samples were used for the zeta potential measurements.

The morphology of the silica beads at each reaction step was analyzed using SEM (TM4000Plus-II; Hitachi High-Tech, Tokyo, Japan).

### Elution behavior of drugs in the bead-packed columns

The elution behavior of 13 drugs that require TDM was investigated to evaluate the performance of the prepared beads as column packing materials.

The prepared beads were suspended in a mixed water–methanol solvent (water:methanol volume ratio of 1:1). Thereafter, the bead suspension was poured into the reservoir of a column packer connected to a stainless-steel column (inner diameter and length of 4.6 and 50 mm, respectively). The beads were packed by flowing the suspension in the mixed water–methanol solvent into the column at a constant pressure of 350 kg/cm^2^ for 1 h. Subsequently, the packed column was connected to an HPLC system (Chromaster; Hitachi High-Tech Science), followed by rinsing the column with pure water for 17 h.

The column performance was evaluated using several hydrophobic steroids. The properties of the steroids are summarized in Table S1. Steroid samples were prepared by dissolving 5 mg of steroids in 1 mL of tetrahydrofuran (THF). Water was then added to a total volume of 5 mL. The solutions were then filtered through a syringe filter. The filtered solutions (100 μL) and pure water (500 μL) were mixed, and the mixed solutions were used as analytes. The elution behavior of steroids in the prepared columns was observed using an HPLC system (Chromaster; Hitachi High-Tech Science). Pure water was used as the mobile phase at a flow rate of 1.0 mL/min. The elution behavior of steroids was observed at a wavelength of 254 nm. The column temperature was controlled using a column oven.

The elution behavior of 13 drugs that require TDM (voriconazole, disopyramide, zonisamide, lidocaine, mexiletine, quinidine, carbamazepine, phenytoin, diazepam, lamotrigine, phenobarbital, mycophenolic acid, and sotalol) was evaluated. Mexiletine and sotalol were dissolved in pure water to obtain 100 μg/mL solutions. The other drugs were dissolved in THF to obtain 100 μg/mL solutions. The elution behavior of the drugs in the prepared columns was evaluated using an HPLC system (LM1010 evaluation device; Hitachi High-Tech Science). The mobile phases comprised 10 mmol/L CH_3_COONH_4_ (pH 4.7) or 10 mmol/L CH_3_COONH_4_ (pH 6.8), and the mobile phase flow rate was set at 1.0 mL/min. The properties of the drugs and their corresponding detection wavelengths are summarized in Tables S2-1 and S2-2. The column temperature was maintained at 30 °C using a column oven.

The serum drug samples were prepared as follows. Mexiletine and sotalol were dissolved in small amounts of pure water. THF was then added to adjust the concentration of the solutions to 100 μg/mL. The other drugs were dissolved in only THF to obtain 100 μg/mL solutions. Each prepared solution (500 μL) was then added to a microtube. The solvent was evaporated under flowing nitrogen gas. Serum solutions were prepared by adding water to freeze-dried serum (Nissui Pharmaceutical Co., Tokyo, Japan). Subsequently, the serum (1.0 mL) was added to each drug-containing microtube. The prepared solutions, which were used as model serum samples, were pretreated before column injection using a solid-phase extraction spin centrifuge column (Hitachi High-Tech Science), according to the manufacturer’s instructions.

Clinical specimens were obtained by collecting serum samples from patients who were administered voriconazole. The human serum samples were pretreated using the same procedure as that used for the model serum samples. The elution behavior of voriconazole in the prepared PN-0 column with the mobile phase comprising 10 mmol/L CH_3_COONH_4_ (pH 4.7) at a flow rate of 1.0 mL/min was observed using an HPLC system (LM1010 evaluation device; Hitachi High-Tech Science). The column temperature was maintained at 30 °C using a column oven. The concentration of voriconazole in the human serum samples was measured using reversed-phase chromatography with an octadecylsilyl column using LM1010 (Hitachi High-Tech Science) with a mobile phase (Hitachi High-Tech Science).

This study was approved by the ethics committee of Keio University (Nos. 210909-3 and 20190218). Informed consent was obtained from all patients. All experiments were performed in accordance with the relevant guidelines and regulations.

## Supplementary Information

Supplementary Information.

## Data Availability

The datasets used and analyzed during the current study are available from the corresponding author upon reasonable request.
