# Supplementary material for: Chromatography columns packed with thermoresponsive-cationic-polymer-modified beads for therapeutic drug monitoring
Source: Sci Rep. 2022 Jul 27;12:12847. doi: 10.1038/s41598-022-16928-3 (PMC9329465; doi:10.1038/s41598-022-16928-3)
Supplement: Supplementary file 1 — Supplementary Information. [file 41598_2022_16928_MOESM1_ESM.docx]

Supplementary Information

**Chromatography columns packed with thermoresponsive-cationic-polymer-modified beads for therapeutic drug monitoring**

Kenichi Nagase^1^*, Hikaru Takagi^1^, Hideo Nakada^1,2^, Haruki Ishikawa^1,2^,

Yoshiko Nagata^1^, Tohru Aomori^1,2^, and Hideko Kanazawa^1^

^1^ Faculty of Pharmacy, Keio University, 1-5-30 Shibakoen, Minato, Tokyo 105-8512, Japan.

^2^ Department of Pharmacy, Keio University Hospital, 35 Shinanomachi, Shinjuku, Tokyo 160-8582, Japan

*Corresponding author: (Phone) +81-3-5400-1378; (E-mail) nagase-kn@pha.keio.ac.jp

**Materials**

*N*-Isopropylacrylamide (NIPAAm) and *N,N*-dimethylaminopropyl acrylamide (DMAPAAm) were provided by KJ Chemicals (Tokyo, Japan). NIPAAm and DMAPAAm were purified via recrystallization from *n*-hexane and distillation, respectively. n-Butyl methacrylate (BMA), *N,N'*-methylene*bis*acrylamide, 4,4-azobis(4-cyanovaleric acid), methanol, ethanol, hydrocortisone, prednisolone, dexamethasone, testosterone, acetic acid, ammonium acetate, carbamazepine, phenytoin, phenobarbital, diazepam, mycophenolic acid, lidocaine, sotalol, and zonisamide were purchased from Fujifilm Wako Chemicals (Osaka, Japan). BMA was purified via distillation. *N*-Ethoxycarbonyl-2-ethoxy-1,2-dihydroquinoline was obtained from the Peptide Institute (Osaka, Japan). N,N-Dimethylformamide and tetrahydrofuran were obtained from Kanto Chemicals (Tokyo, Japan). Hydrocortisone acetate, voriconazole, lamotrigine, disopyramide, quinidine, propafenone hydrochloride, mexiletine, and digoxin were acquired from Tokyo Chemical Industries (Tokyo, Japan). Freeze-dried serum was obtained from Nissui Pharmaceutical Co. (Tokyo, Japan). Aminopropyl silica beads (diameter: 5 μm, pore diameter: 120 Å, Surface Area: 310 m^2^/g) were purchased from YMC (Kyoto, Japan). Stainless-steel columns (inner diameter: 4.6 mmol/L, column length: 50 mmol/L) were purchased from Nishio Kogyo (Tokyo, Japan). A solid-phase extraction spin centrifuge column was purchased from Hitachi High-Tech Science, Tokyo, Japan).

**Table S1.** Properties of hydrophobic steroids.

| Steroid | Structure | Molecular weight | Log*P* | p*K*_a_ |
| --- | --- | --- | --- | --- |
| Hydrocortisone | 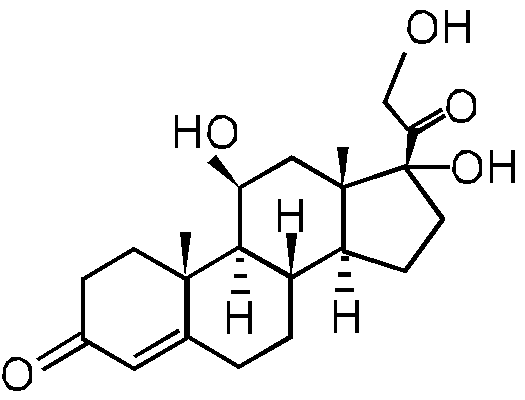 | 362.46 | 1.61 | 12.58 |
| Prednisolone | 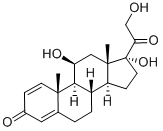 | 360.44 | 1.62 | 12.58 |
| Dexamethasone | 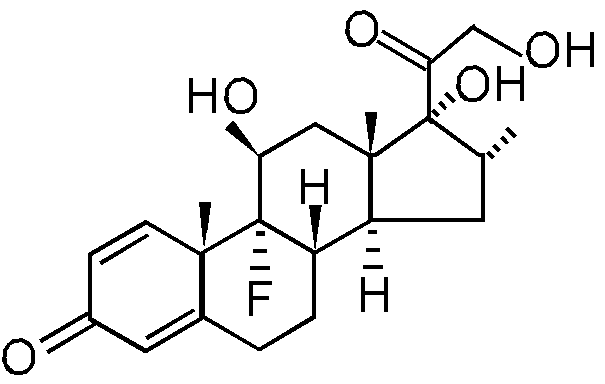 | 392.46 | 1.83 | 13.5 |
| Hydrocortisone acetate | 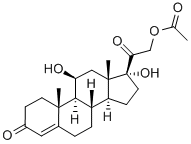 | 404.50 | 2.30 | 12.61 |
| Testosterone | 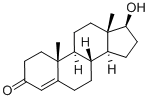 | 288.42 | 3.32 | 15.06 |

^a^Partition coefficients in *n*-octanol–water systems

**
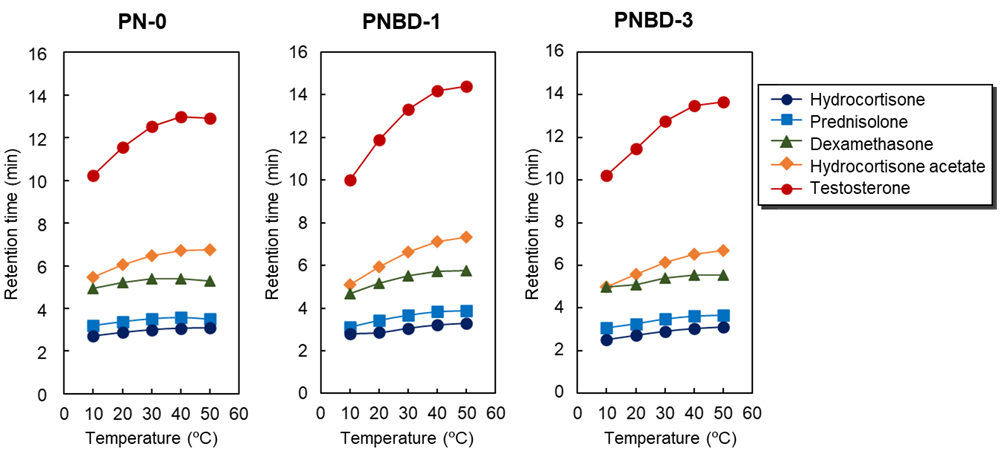
**

**Figure S1.** Temperature dependence of the retention times of several steroids.

**Table S2.1.** Properties of drugs that require therapeutic drug monitoring.

| Classification | Compound | Structure | Molecular weight | Log*P* | p*K*_a_ | Detection wavelength  (nm) |
| --- | --- | --- | --- | --- | --- | --- |
| Antifungal drug | Voriconazole | 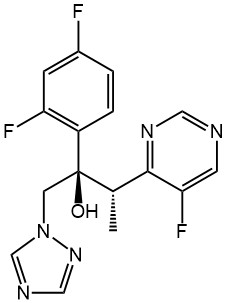 | 349.31 | 1.21 | 11.50 | 250 |
| Antiepileptic drugs | Zonisamide | 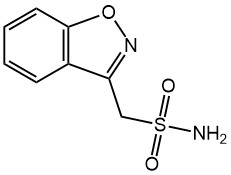 | 212.23 | 0.50 | 9.56 | 280 |
|  | Lamotrigine | 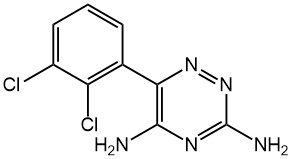 | 256.09 | 2.57 | 5.70 | 310 |
|  | Phenytoin | 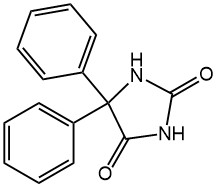 | 252.27 | 2.47 | 8.33 | 210 |
|  | Phenobarbital | 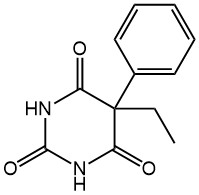 | 232.24 | 1.47 | 7.58 | 240 |
|  | Carbamazepine | 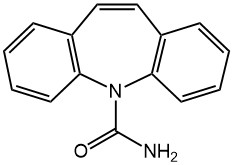 | 236.27 | 2.45 | –3.80, 15.96 | 280 |
|  | Diazepam | 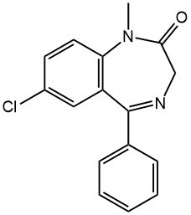 | 284.74 | 2.82 | 3.40 | 240 |

**Table S2.2.** Properties of drugs that require therapeutic drug monitoring.

| Classification | Compound | Structure | Molecular weight | Log*P*^a^ | p*K*_a_ | Detection wavelength  (nm) |
| --- | --- | --- | --- | --- | --- | --- |
| Anti-arrhythmic drugs | Sotalol | 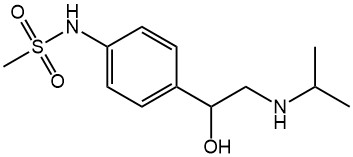 | 272.36 | 0.20 | 8.14, 9.83 | 230 |
|  | Mexiletine | 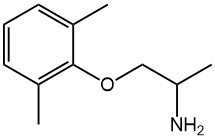 | 179.26 | 2.15 | 8.58 | 220 |
|  | Lidocaine | 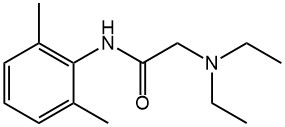 | 234.34 | 2.44 | 7.90 | 220 |
|  | Disopyramide | 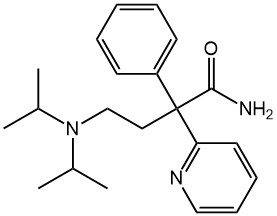 | 339.48 | 2.58 | 10.20 | 260 |
|  | Quinidine | 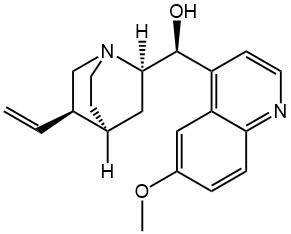 | 324.42 | 3.44 | 8.56 | 235 |
| Immune-suppressing drug | Mycophenolic acid | 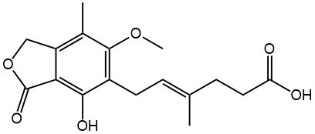 | 320.34 | 2.80 | 5.60 | 300 |

**
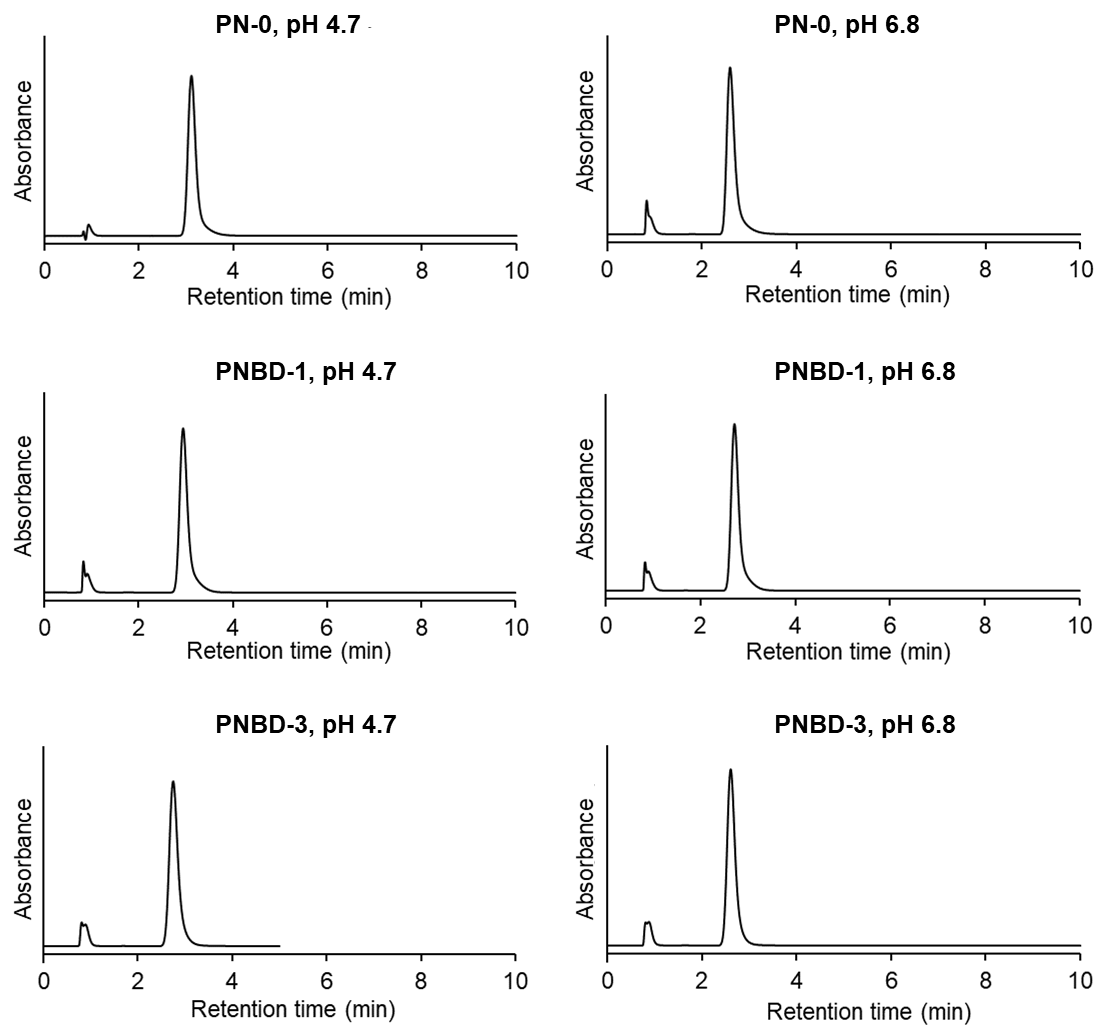
**

**Figure S2.** Chromatograms of voriconazole obtained using the prepared thermoresponsive-cationic-copolymer-hydrogel-modified silica beads. Mobile phase: 10 mmol/L CH_3_COONH_4_ (pH 4.7) or 10 mmol/L CH_3_COONH_4_ (pH 6.8); mobile phase flow rate: 1.0 mL/min; detection wavelength: 250 nm.

**Table S3.** Retention times and peak areas of voriconazole.

| Column | PN-0 | | PNBD-1 | | PNBD-3 | |
| --- | --- | --- | --- | --- | --- | --- |
| pH of  the mobile phase | 4.7 | 6.8 | 4.7 | 6.8 | 4.7 | 6.8 |
| Retention time  (min) | 3.11 | 2.59 | 2.95 | 2.72 | 2.75 | 2.61 |
| Peak area | 1171521 | 1289412 | 1281557 | 1226981 | 1171521 | 1303964 |


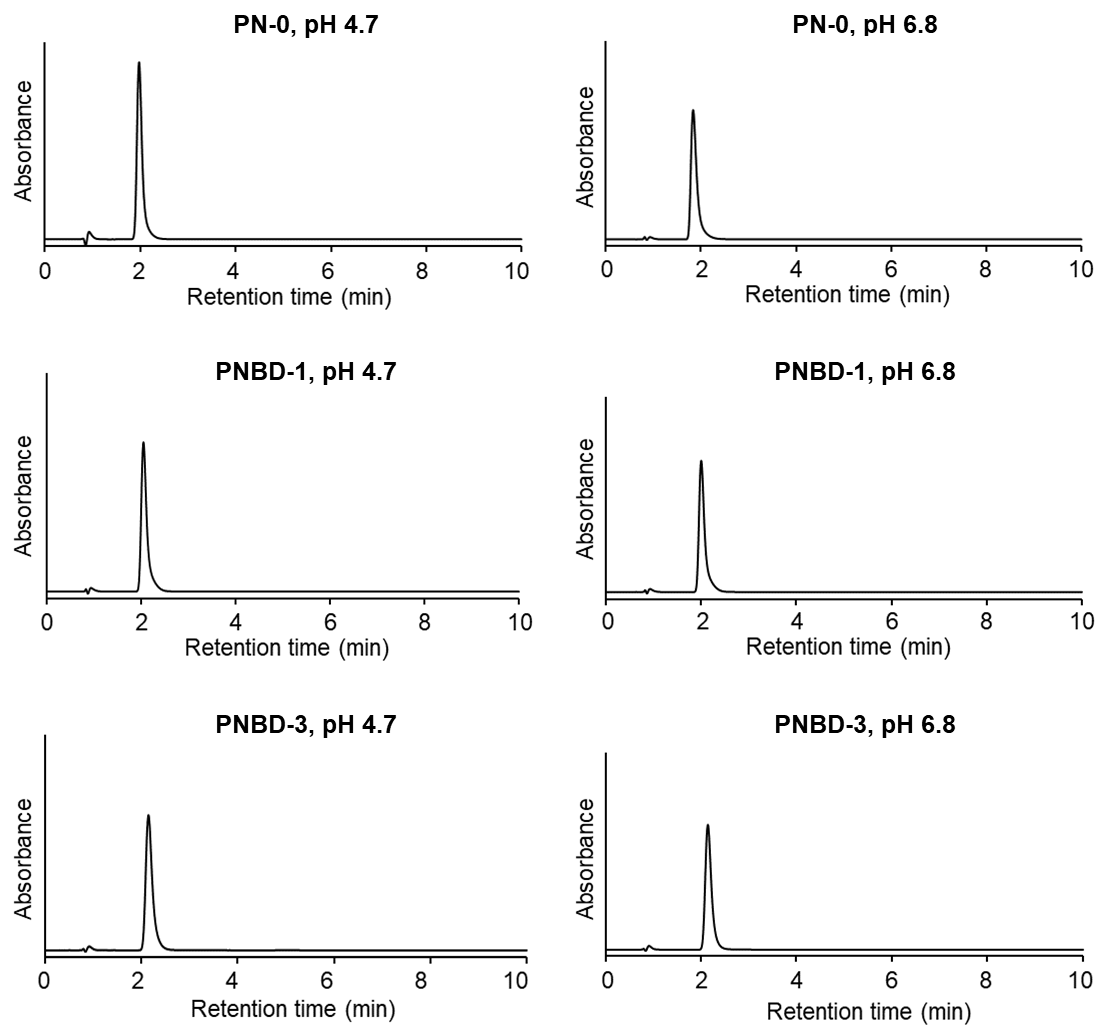


**Figure S3.** Chromatograms of zonisamide obtained using the prepared thermoresponsive-cationic-copolymer-hydrogel-modified silica beads. Mobile phase: 10 mmol/L CH_3_COONH_4_ (pH 4.7) or 10 mmol/L CH_3_COONH_4_ (pH 6.8); mobile phase flow rate: 1.0 mL/min; detection wavelength: 280 nm.

**Table S4.** Retention times and peak areas of zonisamide.

| Column | PN-0 | | PNBD-1 | | PNBD-3 | |
| --- | --- | --- | --- | --- | --- | --- |
| pH of the  mobile phase | 4.7 | 6.8 | 4.7 | 6.8 | 4.7 | 6.8 |
| Retention time  (min) | 1.98 | 1.84 | 2.05 | 2.00 | 2.14 | 2.14 |
| Peak area | 1076061 | 891593 | 886605 | 881363 | 916577 | 881637 |


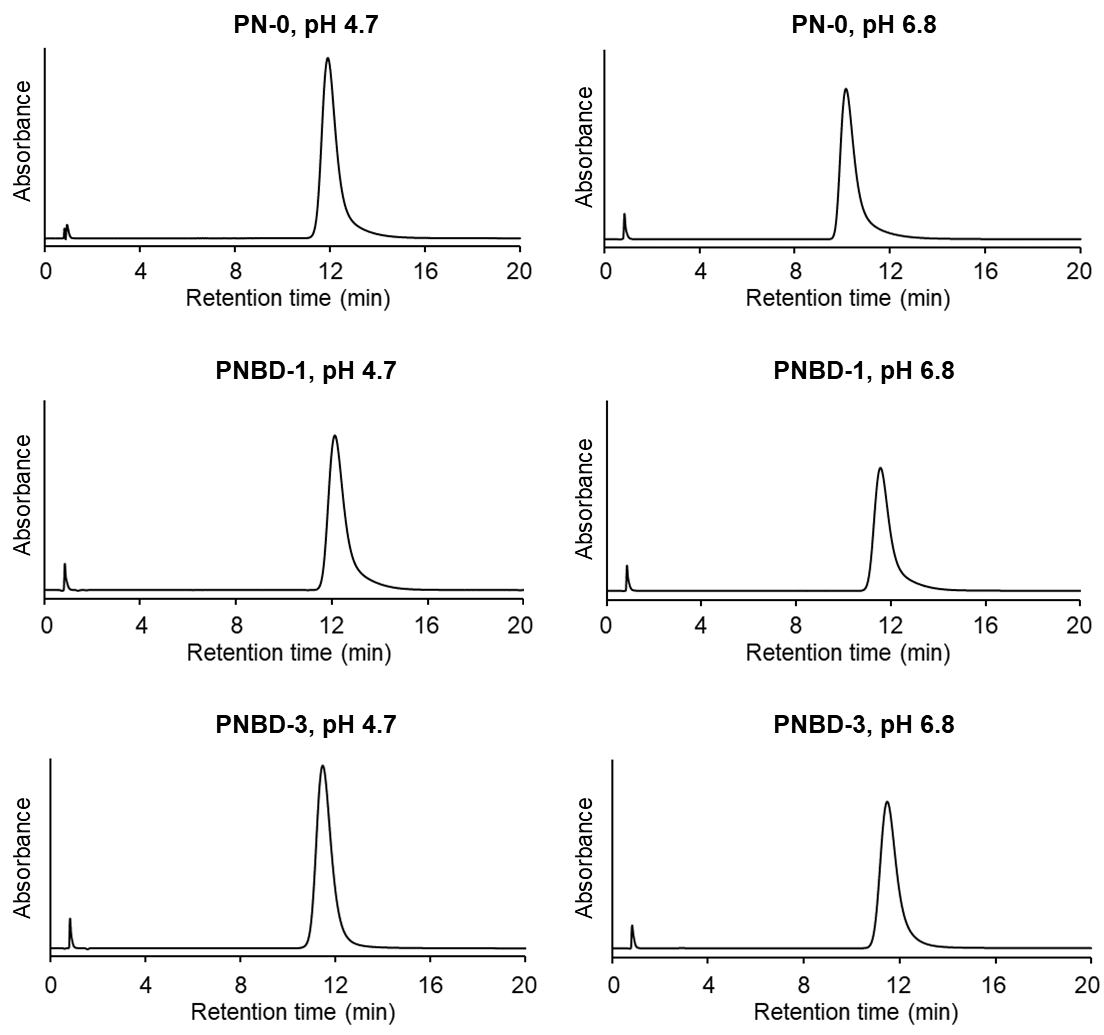


**Figure S4.** Chromatograms of diazepam obtained using the prepared thermoresponsive-cationic-copolymer-hydrogel-modified silica beads. Mobile phase: 10 mmol/L CH_3_COONH_4_ (pH 4.7) or 10 mmol/L CH_3_COONH_4_ (pH 6.8); mobile phase flow rate: 1.0 mL/min; detection wavelength: 240 nm.

**Table S5.** Retention times and peak areas of diazepam.

| Column | PN-0 | | PNBD-1 | | PNBD-3 | |
| --- | --- | --- | --- | --- | --- | --- |
| pH of the  mobile phase | 4.7 | 6.8 | 4.7 | 6.8 | 4.7 | 6.8 |
| Retention time  (min) | 11.91 | 10.15 | 12.18 | 11.57 | 11.45 | 11.48 |
| Peak area | 5075423 | 4063391 | 4537586 | 3204249 | 4821018 | 4272503 |


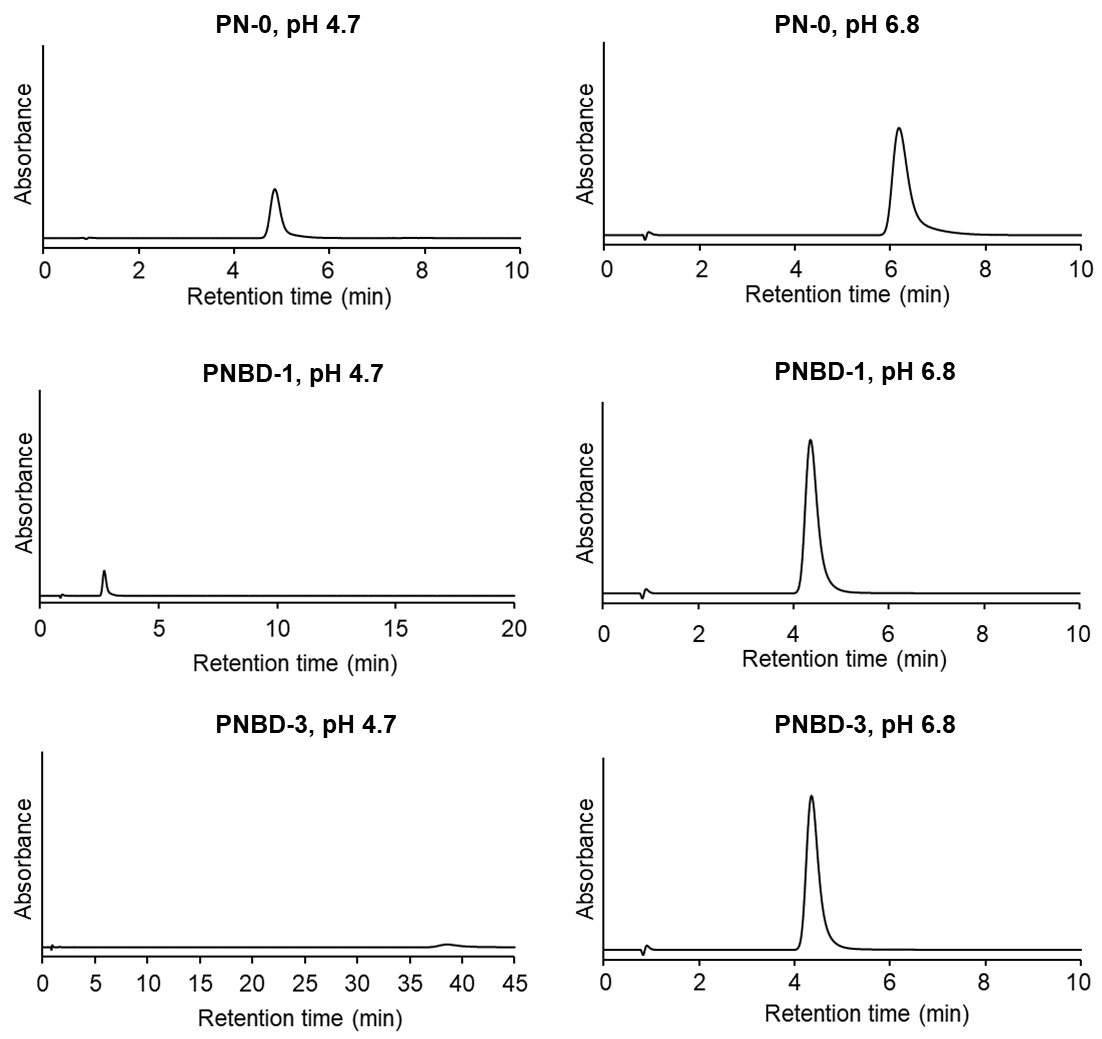


**Figure S5.** Chromatograms of lamotrigine obtained using the prepared thermoresponsive-cationic-copolymer-hydrogel-modified silica beads. Mobile phase: 10 mmol/L CH_3_COONH_4_ (pH 4.7) or 10 mmol/L CH_3_COONH_4_ (pH 6.8); mobile phase flow rate: 1.0 mL/min; detection wavelength: 310 nm.

**Table S6.** Retention times and peak areas of lamotrigine.

| Column | PN-0 | | PNBD-1 | | PNBD-3 | |
| --- | --- | --- | --- | --- | --- | --- |
| pH of the  mobile phase | 4.7 | 6.8 | 4.7 | 6.8 | 4.7 | 6.8 |
| Retention time  (min) | 4.60 | 6.18 | - | 5.32 | 38.57 | 4.36 |
| Peak area | 378806 | 1415295 | - | 1514394 | 192540 | 1513651 |


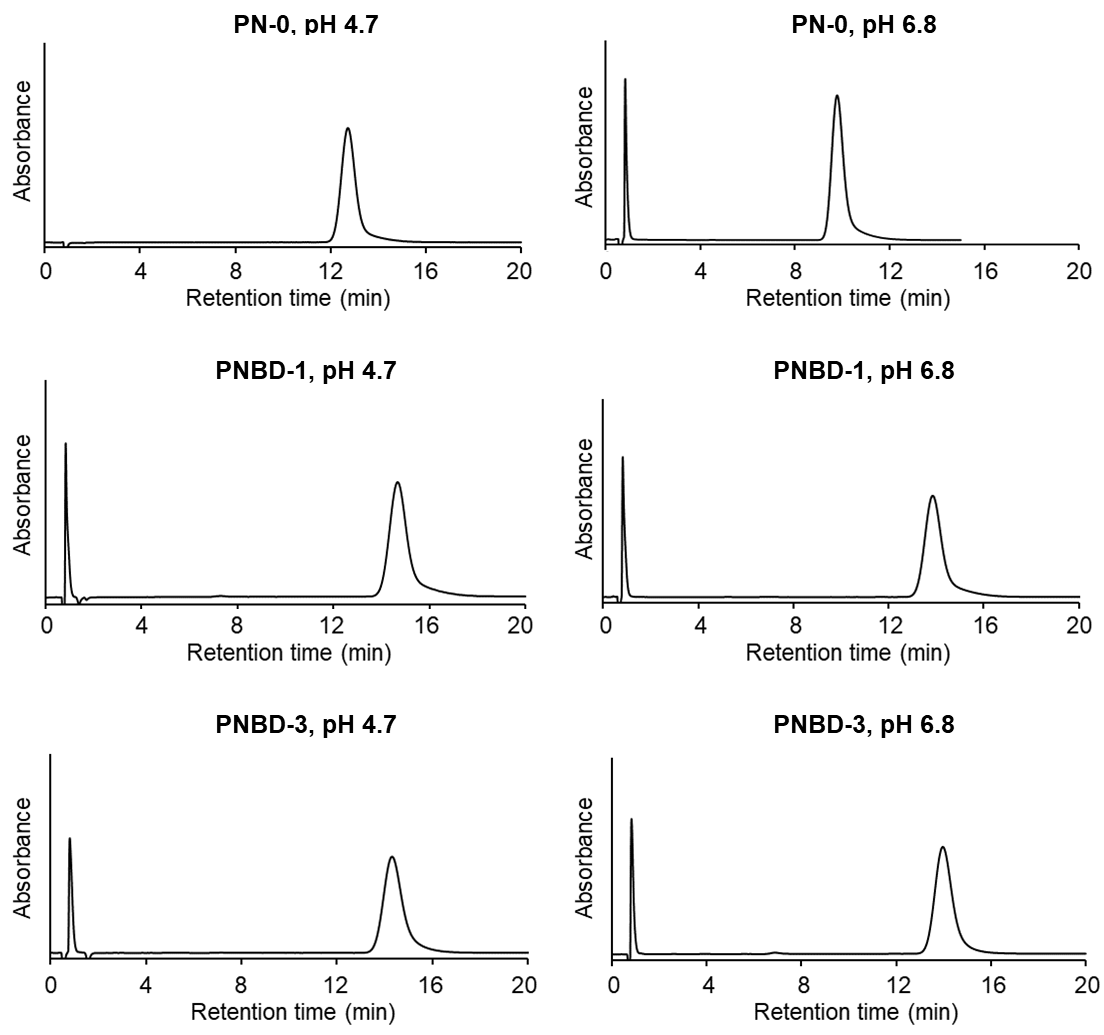


**Figure S6.** Chromatograms of phenytoin obtained using the prepared thermoresponsive-cationic-copolymer-hydrogel-modified silica beads. Mobile phase: 10 mmol/L CH_3_COONH_4_ (pH 4.7) or 10 mmol/L CH_3_COONH_4_ (pH 6.8); mobile phase flow rate: 1.0 mL/min; detection wavelength: 210 nm.

**Table S7.** Retention times and peak areas of phenytoin.

| Column | PN-0 | | PNBD-1 | | PNBD-3 | |
| --- | --- | --- | --- | --- | --- | --- |
| pH of the  mobile phase | 4.7 | 6.8 | 4.7 | 6.8 | 4.7 | 6.8 |
| Retention time  (min) | 11.70 | 9.74 | 14.67 | 13.85 | 14.32 | 13.96 |
| Peak area | 4473309 | 5737263 | 5637804 | 5541706 | 5668450 | 5818301 |


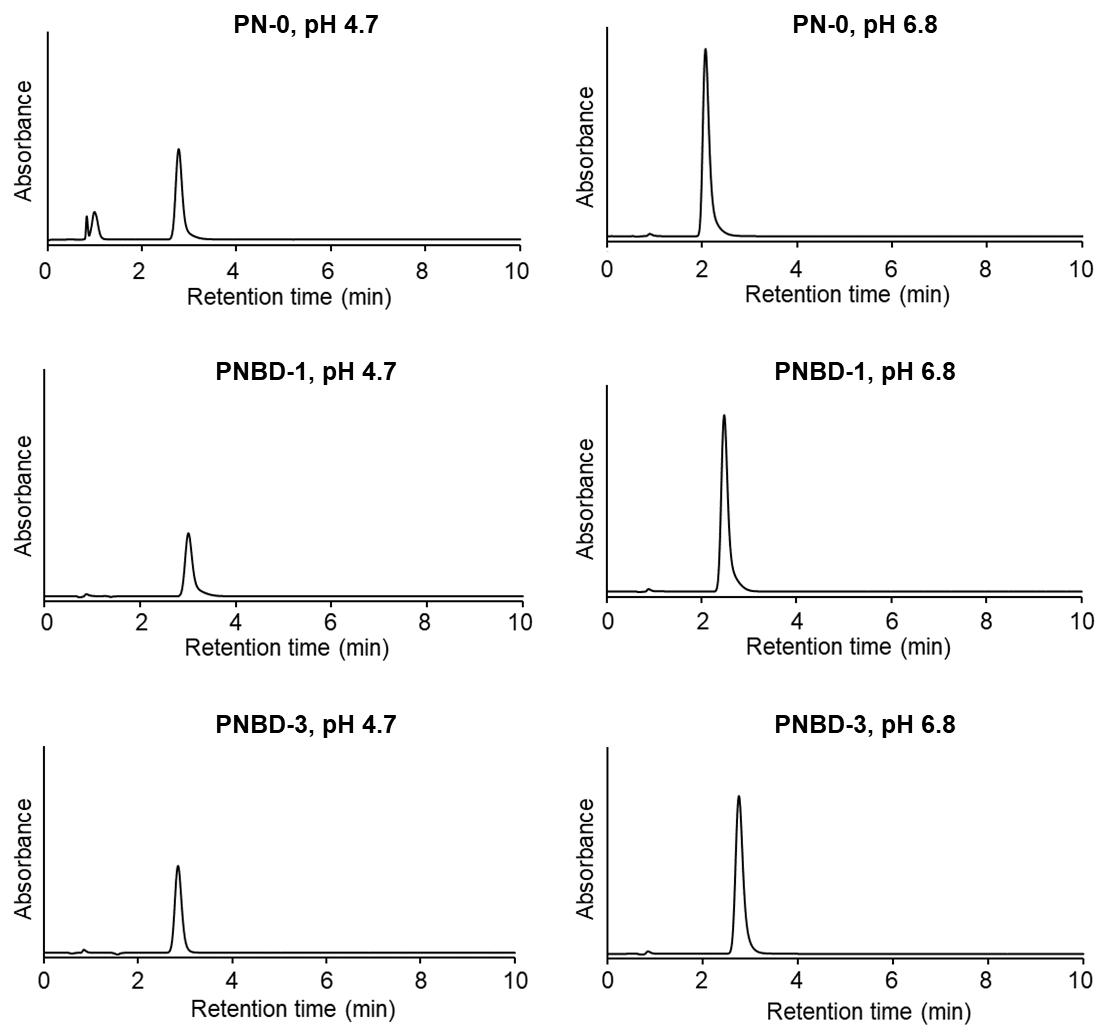


**Figure S7.** Chromatograms of phenobarbital obtained using the prepared thermoresponsive-cationic-copolymer-hydrogel-modified silica beads. Mobile phase: 10 mmol/L CH_3_COONH_4_ (pH 4.7) or 10 mmol/L CH_3_COONH_4_ (pH 6.8); mobile phase flow rate: 1.0 mL/min; detection wavelength: 240 nm.

**Table S8.** Retention times and peak areas of phenobarbital.

| Column | PN-0 | | PNBD-1 | | PNBD-3 | |
| --- | --- | --- | --- | --- | --- | --- |
| pH of the  mobile phase | 4.7 | 6.8 | 4.7 | 6.8 | 4.7 | 6.8 |
| Retention time  (min) | 2.77 | 2.07 | 3.01 | 2.47 | 2.84 | 2.73 |
| Peak area | 373074 | 730688 | 265827 | 549957 | 345247 | 716728 |


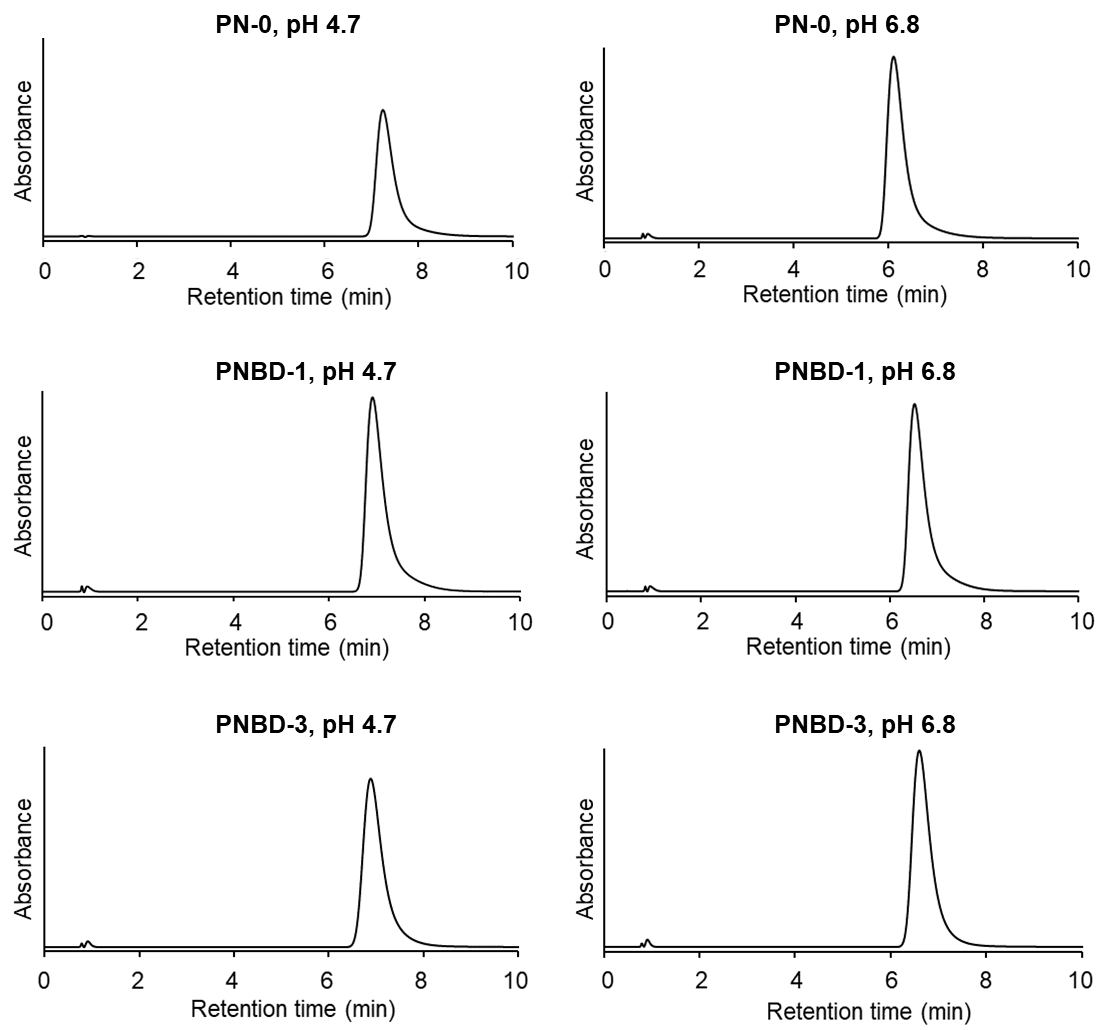


**Figure S8.** Chromatograms of carbamazepine obtained using the prepared thermoresponsive-cationic-copolymer-hydrogel-modified silica beads. Mobile phase: 10 mmol/L CH_3_COONH_4_ (pH 4.7) or 10 mmol/L CH_3_COONH_4_ (pH 6.8); mobile phase flow rate: 1.0 mL/min; detection wavelength: 280 nm.

**Table S9.** Retention times and peak areas of carbamazepine.

| Column | PN-0 | | PNBD-1 | | PNBD-3 | |
| --- | --- | --- | --- | --- | --- | --- |
| pH of the  mobile phase | 4.7 | 6.8 | 4.7 | 6.8 | 4.7 | 6.8 |
| Retention time  (min) | 6.91 | 6.10 | 6.91 | 6.53 | 6.89 | 6.60 |
| Peak area | 2700627 | 5385490 | 5162835 | 5110808 | 5271823 | 5518010 |

**
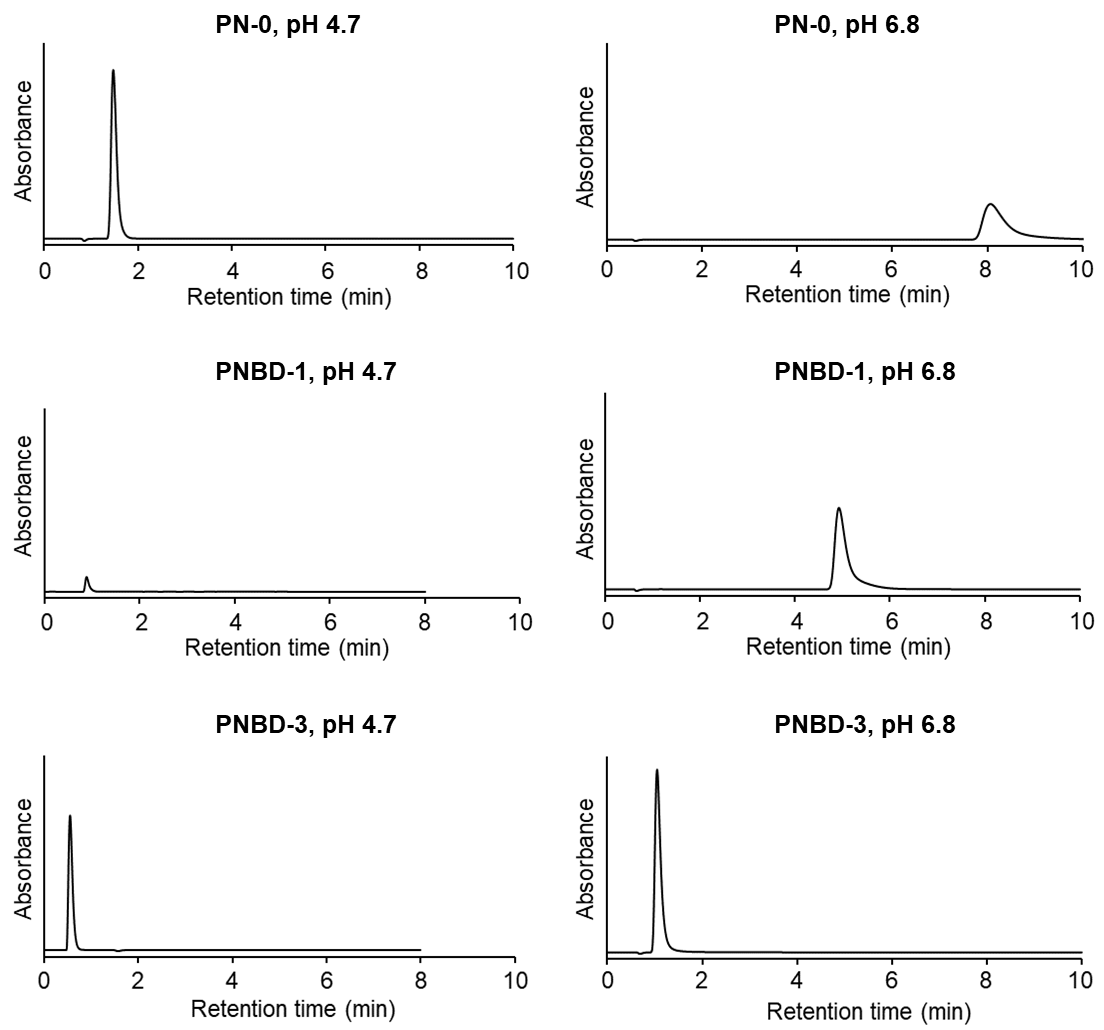
**

**Figure S9.** Chromatograms of sotalol obtained using the prepared thermoresponsive-cationic-copolymer-hydrogel-modified silica beads. Mobile phase: 10 mmol/L CH_3_COONH_4_ (pH 4.7) or 10 mmol/L CH_3_COONH_4_ (pH 6.8); mobile phase flow rate: 1.0 mL/min; detection wavelength: 230 nm.

**Table S10.** Retention times and peak areas of sotalol.

| Column | PN-0 | | PNBD-1 | | PNBD-3 | |
| --- | --- | --- | --- | --- | --- | --- |
| pH of the  mobile phase | 4.7 | 6.8 | 4.7 | 6.8 | 4.7 | 6.8 |
| Retention time  (min) | 1.48 | 8.08 | 0.87 | 4.93 | 0.54 | 1.06 |
| Peak area | 2135348 | 1774558 | 2245085 | 2183554 | 2188637 | 2523598 |

**
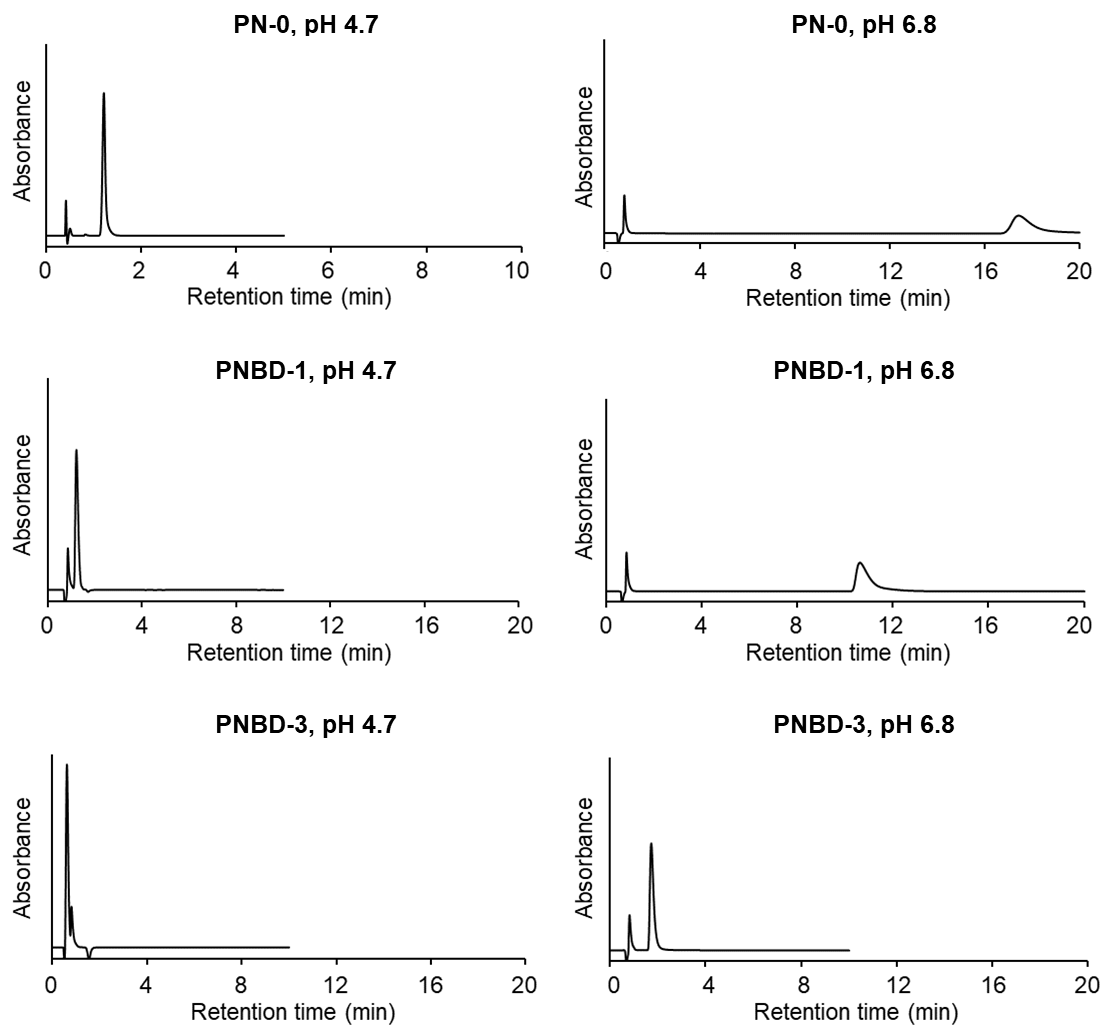
**

**Figure S10.** Chromatograms of mexiletine obtained using the prepared thermoresponsive-cationic-copolymer-hydrogel-modified silica beads. Mobile phase: 10 mmol/L CH_3_COONH_4_ (pH 4.7) or 10 mmol/L CH_3_COONH_4_ (pH 6.8); mobile phase flow rate: 1.0 mL/min; detection wavelength: 220 nm.

**Table S11.** Retention times and peak areas of mexiletine.

| Column | PN-0 | | PNBD-1 | | PNBD-3 | |
| --- | --- | --- | --- | --- | --- | --- |
| pH of the  mobile phase | 4.7 | 6.8 | 4.7 | 6.8 | 4.7 | 6.8 |
| Retention time  (min) | 2.42 | 17.45 | 1.21 | 10.60 | 0.63 | 1.73 |
| Peak area | 1190013 | 881398 | 995487 | 1038568 | 1016076 | 1119103 |


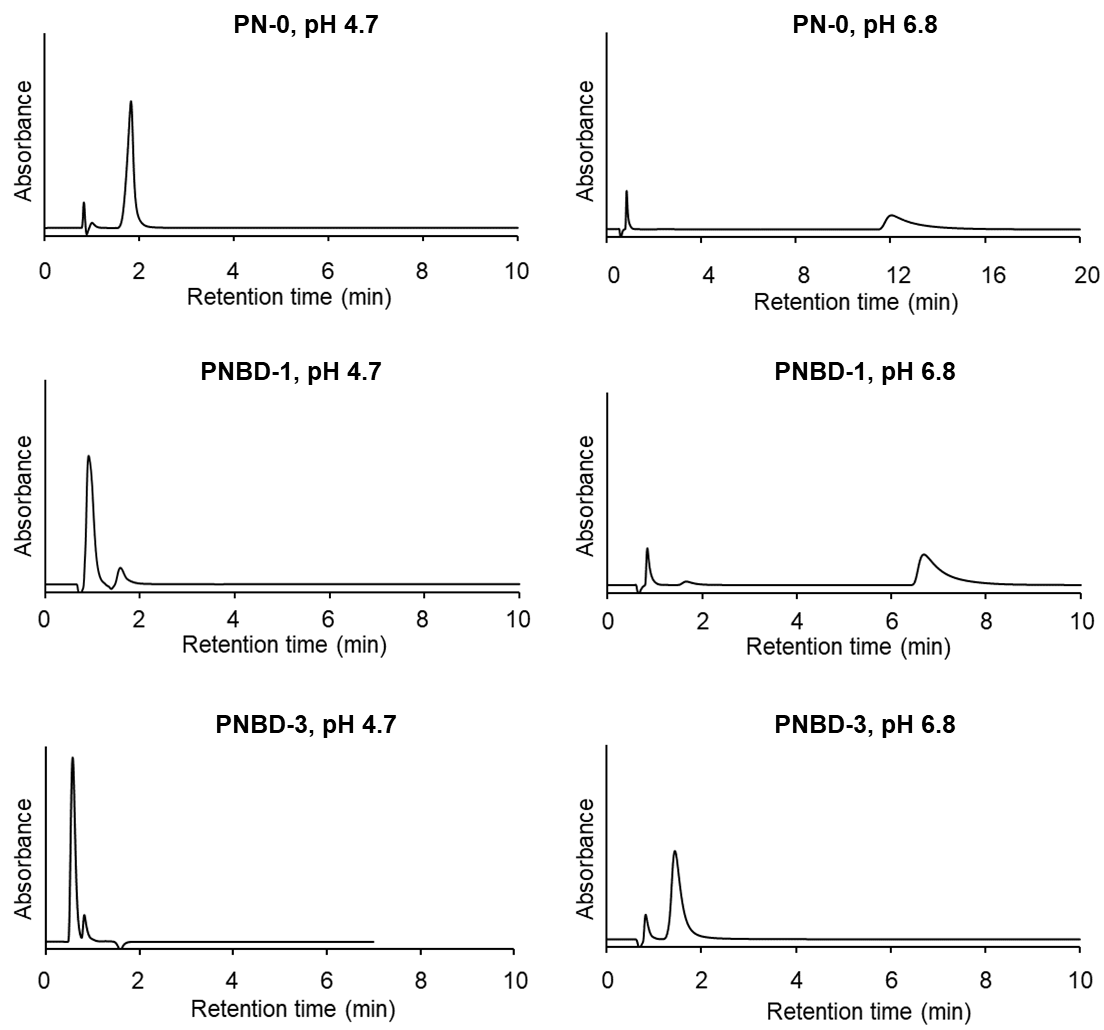


**Figure S11.** Chromatograms of lidocaine obtained using the prepared thermoresponsive-cationic-copolymer-hydrogel-modified silica beads. Mobile phase: 10 mmol/L CH_3_COONH_4_ (pH 4.7) or 10 mmol/L CH_3_COONH_4_ (pH 6.8); mobile phase flow rate: 1.0 mL/min; detection wavelength: 220 nm.

**Table S12.** Retention times and peak areas of lidocaine.

| Column | PN-0 | | PNBD-1 | | PNBD-3 | |
| --- | --- | --- | --- | --- | --- | --- |
| pH of the  mobile phase | 4.7 | 6.8 | 4.7 | 6.8 | 4.7 | 6.8 |
| Retention time  (min) | 1.83 | 11.94 | 0.91 | 6.68 | 0.57 | 1.44 |
| Peak area | 1641604 | 1083096 | 1748980 | 1416551 | 1571823 | 1757311 |

**
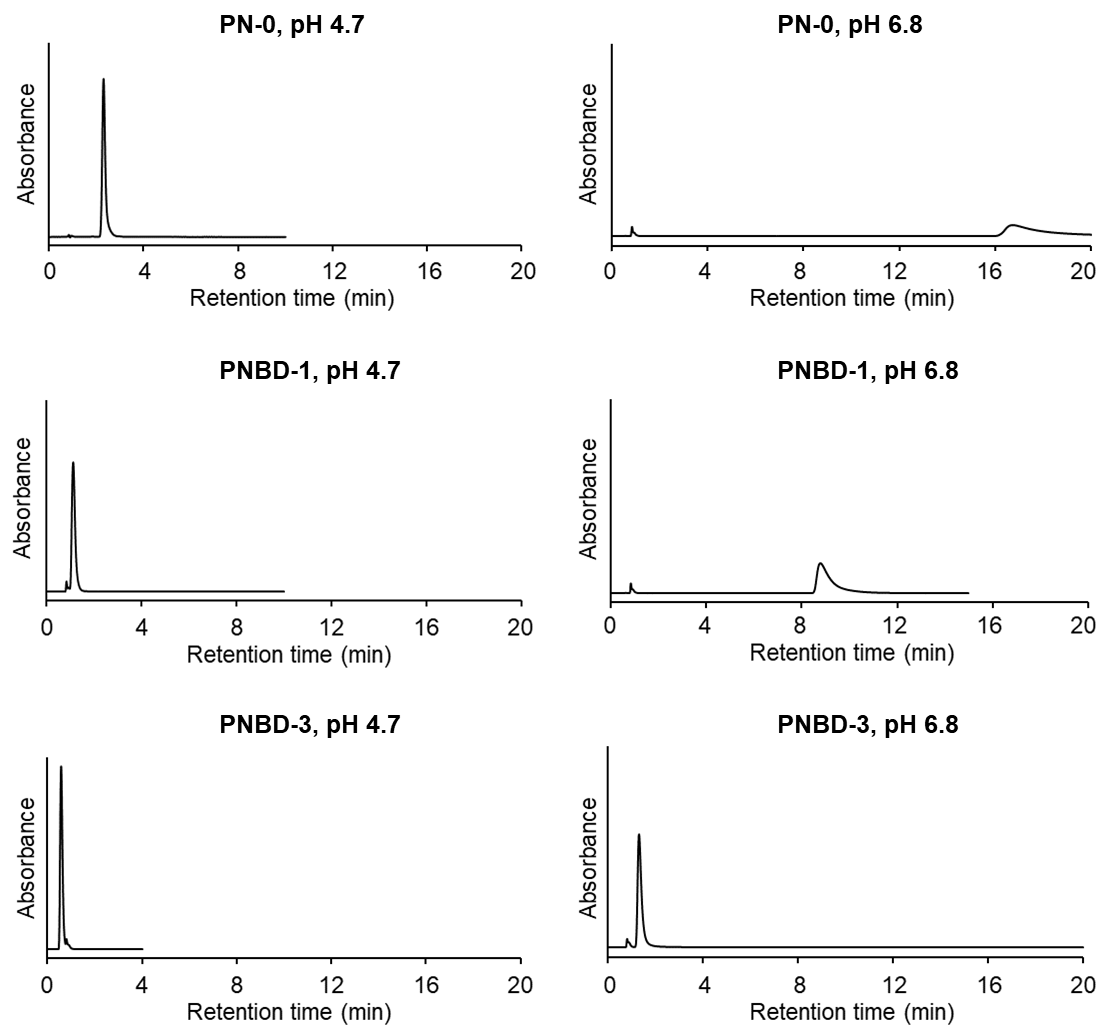
**

**Figure S12.** Chromatograms of disopyramide obtained using the prepared thermoresponsive-cationic-copolymer-hydrogel-modified silica beads. Mobile phase: 10 mmol/L CH_3_COONH_4_ (pH 4.7) or 10 mmol/L CH_3_COONH_4_ (pH 6.8); mobile phase flow rate: 1.0 mL/min; detection wavelength: 260 nm.

**Table S13.** Retention times and peak areas of disopyramide.

| Column | PN-0 | | PNBD-1 | | PNBD-3 | |
| --- | --- | --- | --- | --- | --- | --- |
| pH of the  mobile phase | 4.7 | 6.8 | 4.7 | 6.8 | 4.7 | 6.8 |
| Retention time  (min) | 2.28 | 16.73 | 1.13 | 8.79 | 0.59 | 1.32 |
| Peak area | 879169 | 512694 | 602674 | 639623 | 692731 | 653588 |

**
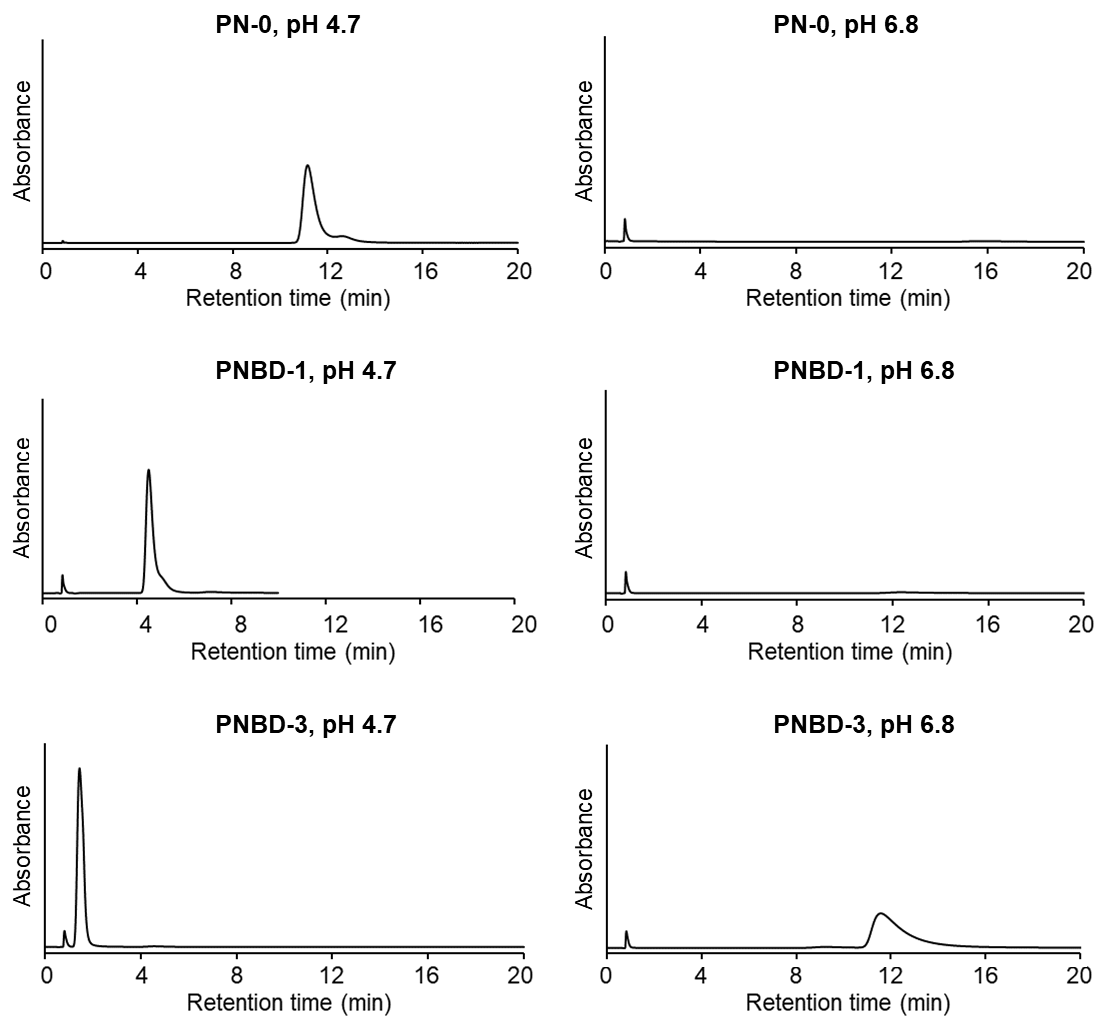
**

**Figure S13.** Chromatograms of quinidine obtained using the prepared thermoresponsive-cationic-copolymer-hydrogel-modified silica beads. Mobile phase: 10 mmol/L CH_3_COONH_4_ (pH 4.7) or 10 mmol/L CH_3_COONH_4_ (pH 6.8); mobile phase flow rate: 1.0 mL/min; detection wavelength: 235 nm.

**Table S14.** Retention times and peak areas of quinidine.

| Column | PN-0 | | PNBD-1 | | PNBD-3 | |
| --- | --- | --- | --- | --- | --- | --- |
| pH of the  mobile phase | 4.7 | 6.8 | 4.7 | 6.8 | 4.7 | 6.8 |
| Retention time  (min) | 10.51 | 15.88 | 4.50 | 12.34 | 1.44 | 11.56 |
| Peak area | 4707208 | 75712 | 5350522 | 248921 | 5593884 | 5431561 |

**
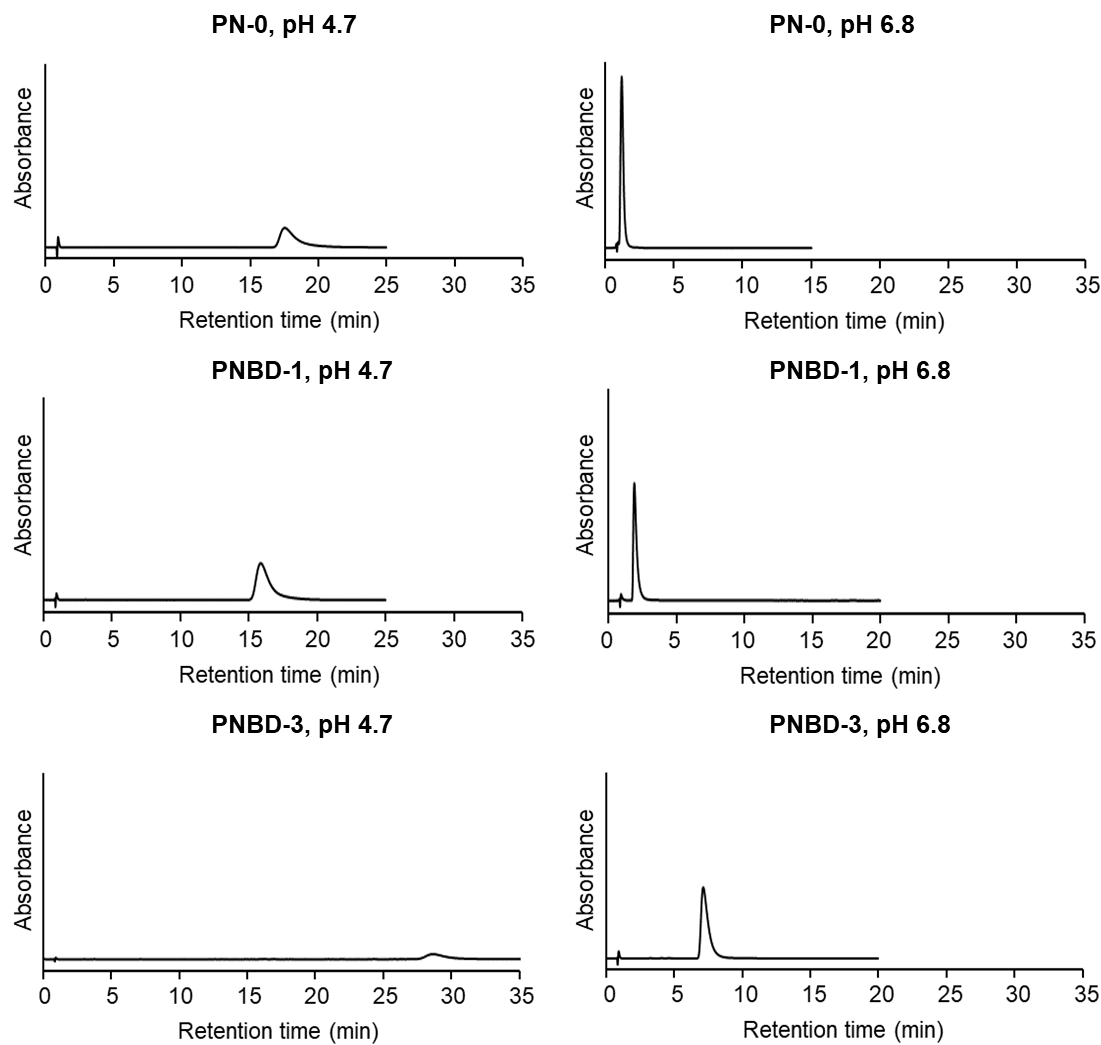
**

**Figure S14.** Chromatograms of mycophenolic acid obtained using the prepared thermoresponsive-cationic-copolymer-hydrogel-modified silica beads. Mobile phase: 10 mmol/L CH_3_COONH_4_ (pH 4.7) or 10 mmol/L CH_3_COONH_4_ (pH 6.8); mobile phase flow rate: 1.0 mL/min; detection wavelength: 300 nm.

**Table S15.** Retention times and peak areas of mycophenolic acid.

| Column | PN-0 | | PNBD-1 | | PNBD-3 | |
| --- | --- | --- | --- | --- | --- | --- |
| pH of the  mobile phase | 4.7 | 6.8 | 4.7 | 6.8 | 4.7 | 6.8 |
| Retention time  (min) | 17.56 | 1.20 | 15.87 | 1.95 | 28.56 | 7.13 |
| Peak area | 718018.7 | 1146024 | 1072323 | 565779 | 288845.7 | 1115073 |

**Table S16.** Optimal measurement conditions for 13 drugs that requires therapeutic drug monitoring.

| Drug | Column | pH of the mobile phase |
| --- | --- | --- |
| Voriconazole | PN-0 | 4.7 |
| Zonisamide | PN-0 | 4.7 |
| Diazepam | PN-0 | 6.8 |
| Lamotrigine | PNBD-3 | 6.8 |
| Phenytoin | PN-0 | 6.8 |
| Phenobarbital | PNBD-3 | 6.8 |
| Carbamazepine | PN-0 | 6.8 |
| Sotalol | PNBD-1 | 6.8 |
| Mexiletine | PN-0 | 4.7 |
| Lidocaine | PN-0 | 4.7 |
| Disopyramide | PN-0 | 4.7 |
| Quinidine | PNBD-1 | 4.7 |
| Mycophenolic acid | PNBD-1 | 6.8 |
